# An Interpretable Ensemble-Learning Framework for Decoding Multi-Component–Multi-Bioactivity Relationships in *Salvia miltiorrhiza* Based on ^1^H NMR Metabolomics and Lifecycle-Oriented Bioassays

**DOI:** 10.3390/metabo16100798

**Published:** 2026-10-21

**Authors:** Wenzhu Li, Shasha Li, Hang Yu, Xiaozhou Zou, Yichao Lu, Zhiyan Chen, Nan Wang, Jiana Shi, Kaiyue Ji, Ping Huang

**Affiliations:** 1 Center for Clinical Pharmacy, Cancer Center, Department of Pharmacy, Zhejiang Provincial People’s Hospital (Affiliated People’s Hospital), Hangzhou Medical College, Hangzhou 310014, China; 2 College of Pharmaceutical Sciences, Zhejiang University, Hangzhou 310058, China; 3 State Key Laboratory of Component-Based Chinese Medicine, Innovation Center in Zhejiang University, Hangzhou 310058, China

**Keywords:** herbal medicine, *Salvia miltiorrhiza* (Danshen), bioassays, AQbD, metabolomics, NMR

## Abstract

**Background**: Linking metabolite composition to biological activities remains challenging in herbal medicines, where diverse metabolites may collectively contribute to pharmacological effects. Although metabolite profiling is fundamental to quality assessment, conventional quality evaluation often provides limited information on how variation in metabolite composition relates to biological activities. **Objectives**: This study aimed to develop a multi-component–multi-bioactivity framework integrating metabolite profiling and bioactivity assessment, enabling bidirectional mapping between metabolite composition and biological responses and providing a functional perspective for quality characterization of complex herbal medicines. *Salvia miltiorrhiza Bunge* (Danshen), a widely used medicinal plant with a chemically diverse metabolome and well-documented cardiovascular activities, was selected as a representative model system. **Methods**: Quantitative ^1^H NMR spectroscopy was employed to profile 28 metabolites in Danshen, while zebrafish-based bioassays were used to assess three cardiovascular-related bioactivities: pro-angiogenic, anti-thrombotic, and vasodilatory activities. A lifecycle-oriented approach was introduced for bioassay development to improve the robustness and reproducibility of biological measurements. Multiple machine learning approaches were integrated within a two-level stacking ensemble framework to construct quantitative metabolite–bioactivity relationship models and capture nonlinear associations between metabolite composition and biological activities. **Results**: The stacked models consistently outperformed the individual learners across the three cardiovascular bioactivities, achieving R^2^ values of 0.895–0.931 and RMSE values of 0.116–0.130. External validation using 10 independent Danshen samples further supported the predictive reliability of the stacked models. Based on model-derived metabolite contributions, a quantitative and interpretable metabolite–bioactivity network linking 28 metabolites with three cardiovascular-related activities was constructed, revealing both shared and activity-specific metabolic contributors. **Conclusions**: This study establishes an interpretable framework integrating NMR-based metabolite profiling, robust multi-endpoint bioassays, and ensemble learning to connect metabolite composition with functional bioactivity. The framework provides a complementary bioactivity-informed perspective for the quality characterization of medicinal plants and supports systems-level evaluation of complex herbal medicines.

## 1. Introduction

Herbal medicines are representative multi-component–multi-bioactivity systems in which therapeutic effects emerge from coordinated interactions among structurally diverse metabolites [1]. Such metabolite complexity poses substantial challenges for both pharmacological interpretation and quality assessment. Although considerable progress has been made in the quality assessment of herbal medicines through strategies such as quality markers (Q-markers) [2] and bioactive equivalent component groups [3], metabolite composition alone provides limited insight into how metabolite variation contributes to pharmacological effects, particularly when multiple metabolites collectively contribute to biological responses [4]. Integrating comprehensive metabolite profiling with quantitative bioactivity assessment is therefore important for establishing metabolite–bioactivity relationships and complementing conventional chemistry-based quality characterization.

Metabolomics has emerged as a powerful strategy for comprehensively characterizing the metabolite composition of herbal medicines, enabling broader assessment of metabolite variation than analyses focused on a limited number of marker compounds [5]. Among metabolomics platforms, quantitative ^1^H nuclear magnetic resonance (^1^H qNMR) spectroscopy offers rapid, reproducible, and non-destructive profiling and simultaneous quantification of structurally diverse metabolites in complex herbal matrices [6,7]. However, metabolomics-based studies of herbal medicines have largely focused on chemical discrimination, authentication, metabolite characterization, and biomarker discovery, whereas the quantitative linkage between metabolite variation and pharmacological activity remains comparatively underexplored [8]. Thus, an important methodological challenge is to move beyond descriptive metabolite profiling toward quantitative integration of metabolic composition with biological phenotypes.

Spectrum–effect relationship studies have been increasingly explored for elucidating the material basis of herbal medicine efficacy [9,10,11]. However, current approaches still suffer from several limitations. Most studies rely primarily on linear statistical methods [12], such as correlation analysis and gray relational analysis, which are insufficient to capture the nonlinear and synergistic interactions inherent to complex herbal systems. In addition, existing models are commonly restricted to associations between single bioactivity and selected secondary metabolites, while neglecting other co-existing metabolites that may contribute to biological responses, resulting in an incomplete representation of the metabolite space. As a result, current spectrum–effect strategies generally lack robust predictive capability and systems-level interpretation of multiple bioactivities. Further elucidation of multi-component–multi-bioactivity relationships therefore requires integrated advances in comprehensive metabolite characterization and nonlinear modeling strategies.

Beyond comprehensive metabolite characterization and nonlinear modeling, reliable bioactivity measurements are equally important for establishing meaningful metabolite–bioactivity relationships. Robust modeling requires biological responses that are sufficiently stable, reproducible, and representative of the pharmacological characteristics of herbal medicines. However, bioassays for herbal medicines are often constrained by complex procedures, high parameter sensitivity, limited standardization, and insufficient reproducibility [13]. Moreover, single-endpoint assays fail to reflect holistic pharmacological effects, whereas multi-endpoint bioassays substantially increase experimental complexity and operational burden [14]. In this context, the Analytical Quality-by-Design (AQbD) framework [15], which has been widely applied in analytical science for systematic method development, risk assessment, and performance control, offers a useful conceptual basis for improving bioassay robustness and supporting lifecycle management [16]. In the present study, AQbD principles were extended to zebrafish-based bioassay development to systematically identify critical experimental parameters and establish robust operating conditions, thereby providing reliable biological measurements for subsequent metabolomics–bioactivity modeling.

**Figure 1:**
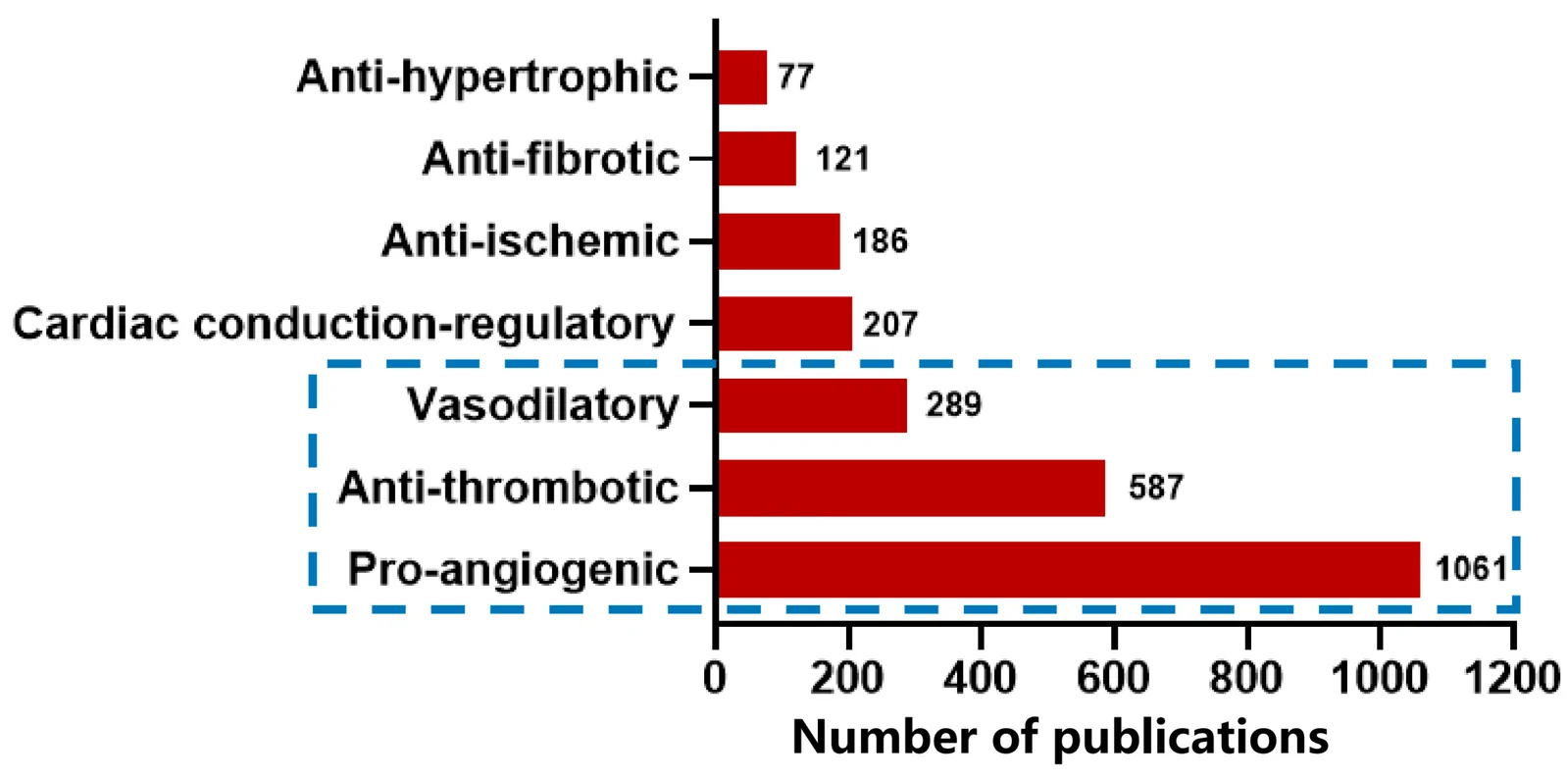
Literature mining of cardiovascular-related bioactivities of Danshen from 2016 to 2026 and selection of representative bioassay endpoints (indicated by the blue dashed-line box). Publication counts are shown for each identified bioactivity.

*Salvia miltiorrhiza Bunge* (Danshen), a widely used medicinal plant in East Asian traditional medicine, was selected as a representative model system [17]. Danshen possesses a chemically diverse metabolome and well-documented cardiovascular activities, making it a suitable model for investigating relationships between metabolite composition and multiple biological effects [18]. Zebrafish have emerged as an advantageous model organism for cardiovascular studies because of their optical transparency and rapid cardiovascular development [19]. Previous studies have demonstrated the successful application of zebrafish models to active constituent screening [20,21], pharmacological evaluation [22,23], and toxicity assessment [24] of Danshen and its bioactive compounds, supporting their suitability as the bioassay platform in the present work. To obtain a representative functional profile of Danshen, the literature mining across multiple scientific databases was performed to identify major cardiovascular-related bioactivities, with details of the search strategy provided in the Supplementary Materials (Section S1). Pro-angiogenic, anti-thrombotic, and vasodilatory activities were subsequently selected as representative biological endpoints (Figure 1). ^1^H qNMR spectroscopy was employed for metabolite profiling because of its suitability for rapid and simultaneous characterization of structurally diverse metabolites in complex herbal systems [25,26]. Our previous studies established the optimized ethanol–water extraction conditions and developed a quantitative ^1^H NMR method for Danshen, providing a methodological basis for the present study [27,28,29]. The combination of quantitative metabolite profiling and multi-endpoint bioactivity assessment thus provides an experimental basis for linking metabolite variation with functional properties of Danshen.

These considerations underscore the need for an integrated approach that can simultaneously address metabolite profiling, multiple bioactivity endpoints, and nonlinear metabolite–bioactivity relationships. Accordingly, this study aimed to develop an interpretable ensemble-learning framework integrating quantitative ^1^H NMR metabolomics, lifecycle-oriented zebrafish bioassays, and nonlinear modeling to decode multi-metabolite–multi-bioactivity relationships in Danshen. The framework combines robust biological measurements with quantitative metabolite profiling to establish bidirectional relationships between metabolite composition and multiple cardiovascular bioactivities, while identifying shared and activity-specific metabolic contributors. By integrating metabolite and bioactivity information, this study provides a bioactivity-informed perspective for the quality characterization of medicinal plants and a scalable strategy for systems-level investigation of complex herbal medicines.

## 2. Materials and Methods

### 2.1. Reagents and Materials

Sixty batches of Danshen were obtained from Zhejiang Tiandao Pharmaceutical Co., Ltd. (Hangzhou, China), with the batch numbers and geographical origins of the samples provided in Table S1. Pronase E (B2517143) and aspirin (A2509078) were obtained from Aladdin Chemical Co., Ltd. (Shanghai, China). Phenylhydrazine (PHZ, C15090360), o-dianisidine (C15678243), anhydrous ferric chloride (FeCl_3_, C15600951), and lenvatinib (C15327639) were purchased from Macklin Biochemical Co., Ltd. (Shanghai, China). Salvianic acid A (DSS, 230930) and lithospermic acid (LA, 231009) were obtained from Shanghai Winherb Medical Technology Co., Ltd. (Shanghai, China).

Deuterium oxide (D_2_O, 99.9 atom% D) containing 3-(trimethylsilyl) propionic-2,2,3,3-d_4_ acid sodium salt (TSP, 0.05 wt%) was purchased from Sigma-Aldrich (St. Louis, MO, USA). Methanol-d_4_ (CD_3_OD, 99.8 atom% D) containing 0.03% (*v*/*v*) tetramethylsilane (TMS) was obtained from Cambridge Isotope Laboratories, Inc. (Andover, MA, USA). Sodium chloride, magnesium sulfate, potassium chloride, calcium chloride, dimethyl sulfoxide (DMSO) and ethanol were of analytical grade. Ultrapure water was used throughout the study.

### 2.2. Sample Preparation

The dried roots of Danshen were pulverized and sieved through a 50-mesh screen. Based on our previous study, in which the ethanol–water extraction conditions were systematically optimized, 75% (*v*/*v*) ethanol was selected as the extraction solvent for the present study [27]. Samples were extracted at a solvent-to-material ratio of 10:1 (*v*/*w*) by ultrasonication for 1 h per extraction. The extraction was performed twice, and the supernatants were combined. The combined extracts were centrifuged at 4400 rpm for 10 min, and the supernatant was collected and aliquoted (1 mL) into 2 mL centrifuge tubes. The aliquots were concentrated under reduced pressure at 50 °C to remove the solvent. Each tube yielded a dried extract equivalent to 50 mg of crude herbal material. The dried extracts were sealed and stored at −80 °C until use for bioactivity assays and NMR analysis.

### 2.3. Zebrafish Husbandry

Wild-type AB and Tg (fli1:EGFP) zebrafish were obtained from the Laboratory Animal Center of Zhejiang University (Hangzhou, China). Adult male and female zebrafish were maintained in a temperature- and light-controlled aquaculture system under a 14 h light/10 h dark photoperiod at 28 ± 0.5 °C and fed brine shrimp twice daily and dry flake feed once daily.

Embryos were generated by natural spawning and maintained in E3 medium (0.286 g/L NaCl, 0.0126 g/L KCl, 0.048 g/L CaCl_2_, and 0.081 g/L MgSO_4_; pH 6.8–7.2). To maintain optical transparency, 1-phenyl-2-thiourea (PTU) was added to the medium. Embryos were staged and used between 24 and 72 h post-fertilization (hpf).

### 2.4. Cardiovascular Bioassays Based on Zebrafish Models

Three zebrafish cardiovascular injury models were established to evaluate the pro-angiogenic, anti-thrombotic, and vasodilatory activities of Danshen extracts: a lenvatinib-induced intersegmental vessel (ISV) injury model (Model 1), a PHZ-induced thrombosis model (Model 2), and a FeCl_3_-induced vascular stenosis model (Model 3).

For Model 1, Tg (fli1:EGFP) embryos at 24 hpf were dechorionated with 1 mg/mL Pronase E and transferred to 24-well plates containing E3 medium supplemented with PTU (10 embryos per well). Embryos were randomly assigned to vehicle control, Lenvatinib model groups (100–600 ng/mL), positive control (LA, 10 μg/mL), and Danshen treatment groups (25–175 μg/mL). Lenvatinib exposure was performed from 24 to 48 hpf, followed by Danshen treatment from 48 to 72 hpf. At 72 hpf, embryos were imaged using a fluorescence stereomicroscope (AxioZoom.V16, ZEISS, Jena, Germany). Angiogenesis-related indices were derived from ISV and body length measurements using Equations (S1.1)–(S1.3).

For Model 2, wild-type AB embryos at 48 hpf were dechorionated and exposed to PHZ (0.78–2.73 nmol/mL) from 48 to 72 hpf, followed by Danshen treatment from 72 to 96 hpf. Aspirin (5 μg/mL) was used as the positive control. At 96 hpf, embryos were stained with 0.6 mg/mL o-dianisidine for 15 min and washed three times with DMSO. Thrombosis-related indices were derived from cardiac erythrocyte staining intensity using Equations (S2.1) and (S2.2).

For Model 3, Tg (fli1:EGFP) embryos at 72 hpf were exposed to FeCl_3_ (5–20 μg/mL) together with Danshen extracts for 24 h, with DSS (10 μg/mL) used as the positive control. At 96 hpf, embryos were imaged under a fluorescence stereomicroscope, and vasodilation-related indices were derived from caudal artery diameter measurements using Equations (S3.1)–(S3.3). All experiments were performed in duplicate. Phenotypic measurements and image analyses were conducted in a blinded manner.

### 2.5. AQbD-Based Bioassay Development

An AQbD framework was applied for the development and optimization of zebrafish-based cardiovascular bioassays. An Ishikawa-based risk assessment supported by literature data, preliminary experiments, and expert knowledge was conducted to identify potential critical bioassay procedure parameters (CBPPs), which were subsequently evaluated, screened, and optimized using a design of experiments (DoE) strategy. High-risk CBPPs were screened using Plackett–Burman designs, followed by optimization using response surface methodology, including central composite designs (CCDs) and I-optimal designs. Experimental responses were defined by critical quality attributes (CQAs), and quantitative relationships between CBPPs and CQAs were established using partial least squares (PLS) regression.

Design spaces were established based on the optimized models using deterministic constraints and Monte Carlo simulation, with simulation parameters provided in Table S2. The design space was defined as the region in which the predefined CQA acceptance criteria were met with a failure probability below 5%. A feasible operating range within the design space was selected as the method operable design region (MODR) for subsequent experimental application.

### 2.6. Validation of Bioassay Methods

Method validation was performed by selecting ten parameter combinations within the MODR to experimentally verify whether the predicted responses met the predefined acceptance criteria. In addition, five batches of Danshen from different origins with substantial differences in metabolite composition were tested using the established bioactivity assay methods. Variations in biological responses among samples were analyzed to evaluate the method’s ability to discriminate composition-dependent bioactivity differences. Statistical analysis was conducted using analysis of variance (ANOVA) followed by Fisher’s post hoc test. Prior to analysis, normality and homogeneity of variance were verified to ensure statistical validity.

### 2.7. NMR Spectroscopy

Due to the broad polarity range of Danshen constituents, a dual-solvent strategy using 90% H_2_O/10% D_2_O and CD_3_OD was applied to ensure comprehensive detection of hydrophilic and lipophilic metabolites, yielding type A and type B samples, respectively.

For type A samples, extracts were dissolved in 540 μL deionized water and 60 μL D_2_O containing 0.05% (*w*/*w*) TSP, vortexed (1500 rpm, 10 min), centrifuged (10,000 rpm, 15 min), and the supernatant was transferred for NMR analysis. For type B samples, residues were washed to remove hydrophilic metabolites, dried under vacuum (40 °C, 30 min), and dissolved in CD_3_OD containing 0.03% TMS.

All samples were analyzed using the standard noesygppr1d pulse sequence on a Bruker AVANCE III 600 MHz NMR spectrometer (Bruker BioSpin GmbH, Rheinstetten, Germany). Acquisition parameters for type A samples were spectral width 11 ppm, transmitter frequency offset 4.696 ppm, relaxation delay 20 s, mixing time 50 ms, 90° pulse width 14.75 μs, dummy scans 4, number of scans 64, acquisition time 2.980 s, and receiver gain 40.3. For type B samples, the transmitter frequency offset was set to 4.908 ppm with receiver gain adjusted to 57, while all other acquisition parameters were identical to those used for type A samples.

### 2.8. Quantitative Analysis of the Metabolites in Danshen Extracts

^1^H NMR spectra were processed using Bruker TopSpin (v3.2.6) and MestReNova (v14.0.0), including exponential apodization (0.30 Hz line broadening), manual phase correction, and automatic baseline correction. Chemical shifts were referenced to TSP/TMS (δ = 0.00 ppm) for spectral alignment. After complete assignment of characteristic resonances, constituent quantification was carried out using a previously developed ^1^H qNMR method [27,28,29], with TSP/TMS as the internal standard calibration, as described in Equation (S4).

### 2.9. Data Preprocessing and Statistical Analysis

A multi-dimensional metabolite–bioactivity relationship framework was established by integrating chemometric and ensemble machine learning methods. Among the 60 batches of Danshen samples, 50 batches were randomly assigned to the training set and the remaining 10 batches were reserved as an independent external validation set. The predictor matrix (X) consisted of quantitative profiles of 28 metabolites obtained by ^1^H qNMR analysis, while the response matrix (*Y*) contained three cardiovascular bioactivity indices corresponding to pro-angiogenic, anti-thrombotic, and vasodilatory activities. Prior to modeling, predictor variables were standardized using z-score normalization.

PLS regression, support vector regression (SVR), random forest (RF), and gradient boosting (LSBoost) models were constructed to characterize multi-dimensional relationships between metabolite composition and cardiovascular bioactivities. A two-level stacking ensemble framework was further developed by integrating predictions from SVR, RF, and LSBoost using a linear regression meta-model (Equation (S7)). To minimize information leakage, five-fold cross-validation was performed within the training set to generate out-of-fold (OOF) predictions from the three base learners. The OOF predictions were then used to train the meta-regression model. Subsequently, the base learners were refitted using the entire training set, and their predictions for the independent external validation set were combined using the trained meta-model to obtain the final ensemble predictions. Variable importance was evaluated based on RF permutation importance and LSBoost predictor importance, followed by normalized integration analysis. Bootstrap resampling (100 iterations) was applied to estimate prediction uncertainty and derive 95% confidence intervals. All analyses were performed in MATLAB R2022b (MathWorks, Natick, MA, USA).

## 3. Results

### 3.1. AQbD-Based Development of a Multi-Index Cardiovascular Bioassay for Danshen

#### 3.1.1. Initial Establishment of Zebrafish Models

**Figure 2:**
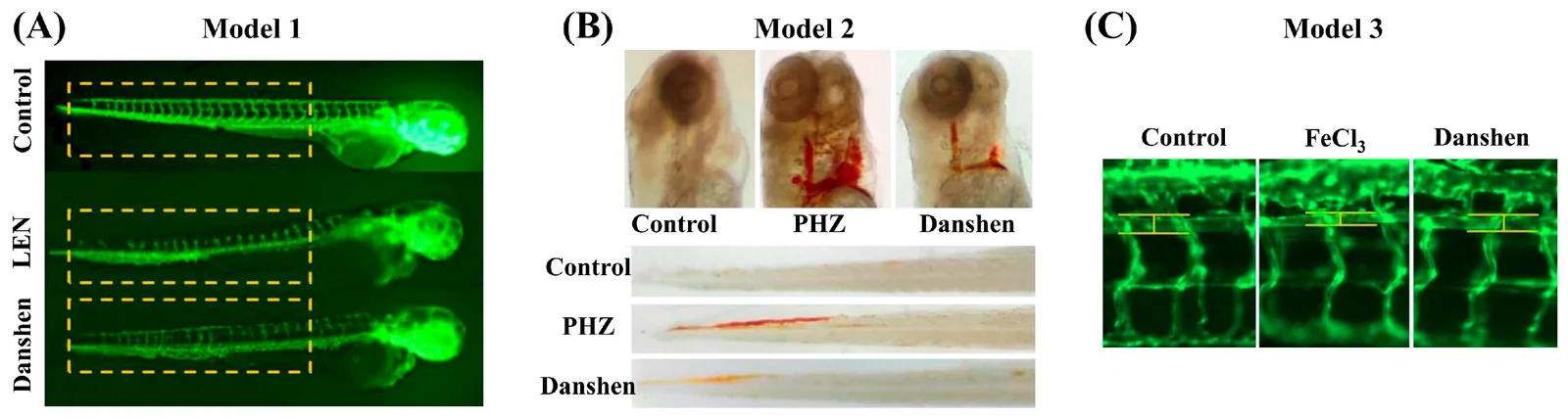
Representative images of the ISVs injury model (**A**), thrombosis model (**B**), and vascular stenosis model (**C**).

Three complementary pathological zebrafish models were developed to characterize distinct aspects of cardiovascular injury, including angiogenesis impairment, thrombosis formation, and vascular stenosis. In the ISVs injury model (Model 1), Lenvatinib (LEN) induced a concentration-dependent loss of ISVs, whereas Danshen treatment markedly restored vascular integrity, indicating pro-angiogenic activity (Figure 2A). In the thrombosis model (Model 2), PHZ exposure led to extensive thrombus formation, which was significantly reduced following Danshen intervention (Figure 2B). In the vascular stenosis model (Model 3), FeCl_3_ treatment decreased caudal artery diameter relative to the control, while Danshen partially reversed this effect (Figure 2C). These results demonstrate the feasibility of the three models for multi-endpoint evaluation of the cardiovascular effects of Danshen.

In addition, preliminary range-finding experiments further defined the working concentration ranges of modeling agents and Danshen extracts (Figure S1), establishing initial boundaries for CBPPs and CQAs to support subsequent AQbD-driven optimization.

#### 3.1.2. Risk Analysis

**Figure 3:**
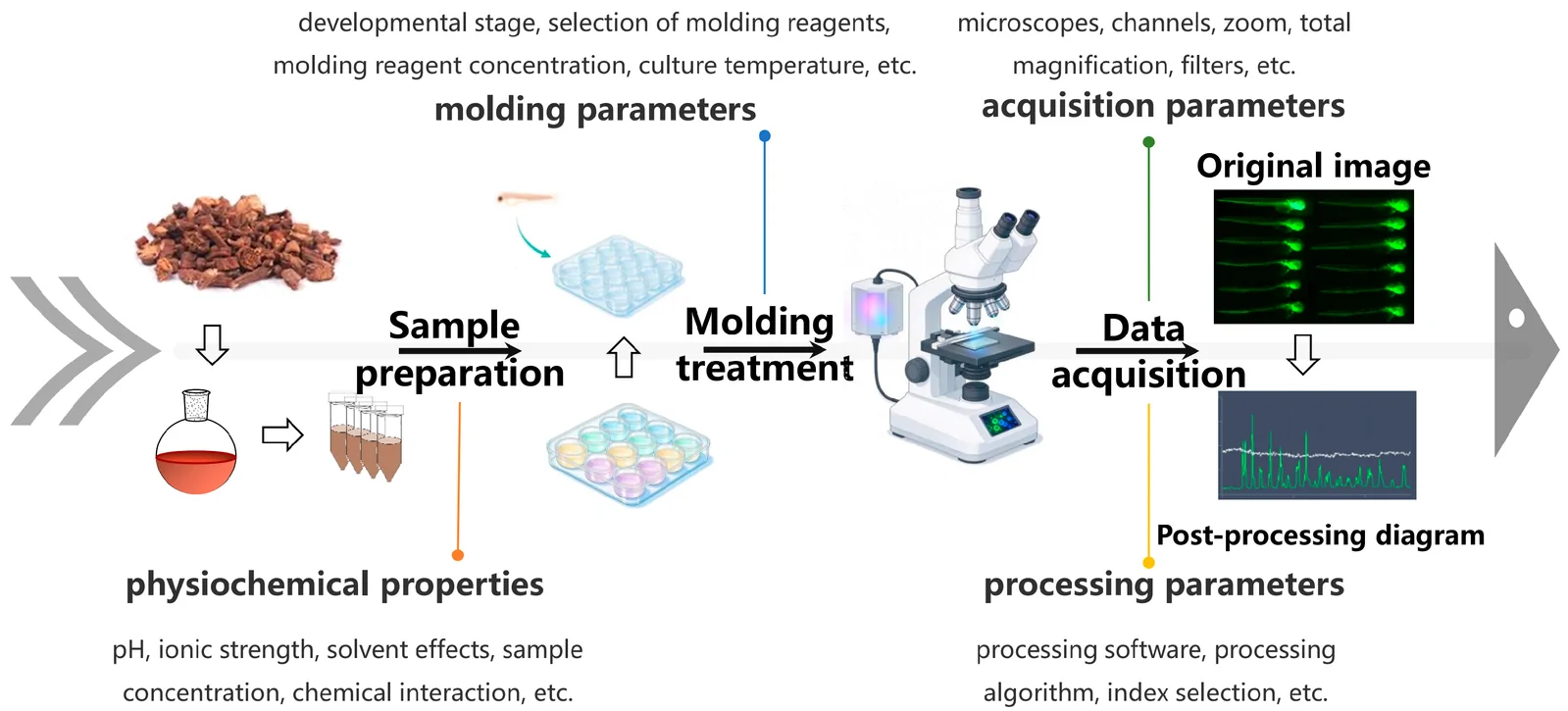
Ishikawa diagram-based risk assessment of the bioassay for Danshen.

Building on the three zebrafish models established in Section 3.1.1, bioassay methods were further developed for the evaluation of pro-angiogenic, anti-thrombotic, and vasodilatory activities of Danshen. In accordance with the objectives of the bioassays, CQAs were defined for each bioassay and are summarized in Table 1. Of these, *R*_13_, *R*_14_, *R*_23_, *R*_24_, *R*_33_, and *R*_34_ were defined as supplementary CQAs exclusively for optimization purposes, while the remaining CQAs were employed in both screening and optimization stages. Based on prior knowledge and experimental considerations, an Ishikawa diagram (Figure 3) was employed to systematically identify and rank factors potentially affecting the CQAs. The assessed variables encompassed physicochemical properties, modeling conditions, signal acquisition, and data processing. This risk assessment identified 18 potential CBPPs, as summarized in Table 1, providing the basis for subsequent DoE-based screening and optimization.

#### 3.1.3. Screening of CBPPs Based on Plackett–Burman Design

Based on the risk analysis, PB designs were applied to screen CBPPs for the zebrafish-based bioassay methods. The 18 potential CBPPs were defined as factors for PB screening, while *R*_11_, *R*_12_, *R*_21_, *R*_22_, *R*_31_, and *R*_32_ were included as response variables (Table 1). A total of 36 experimental runs were performed, with 12 runs for each bioactivity assay. The PB design matrices, coded factor levels, and experimental results for the three bioactivity assays are presented in Tables S3.1–S3.3, respectively.

**Figure 4:**
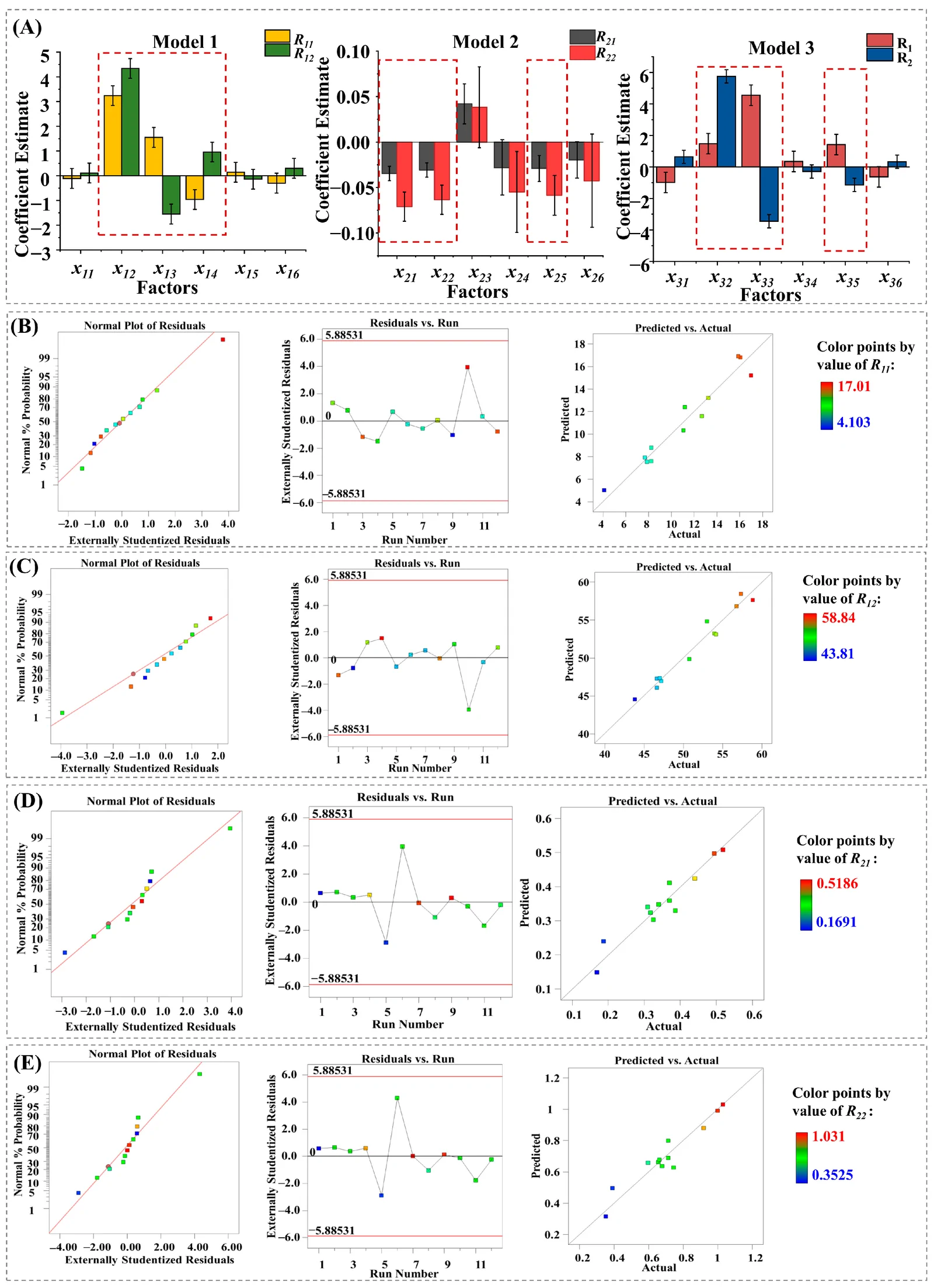

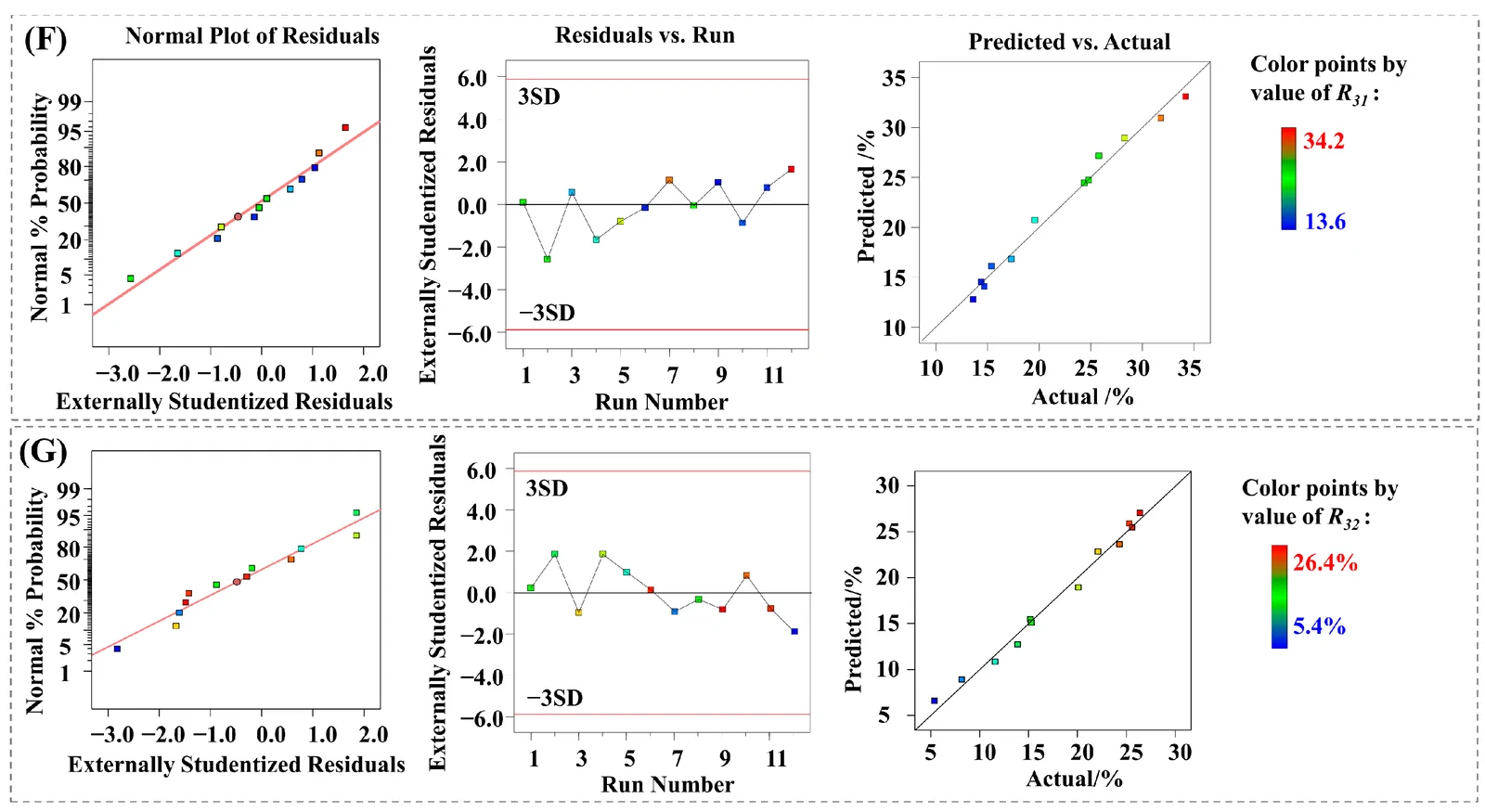
(**A**) Coefficient estimates of the 18 factors obtained from the Plackett–Burman design; (**B**–**G**) model diagnostics for the six response variables, including normal probability distributions, residual plots, and predicted versus observed values for *R*_11_ (**B**), *R*_12_ (**C**), *R*_21_ (**D**), *R*_22_ (**E**), *R*_31_ (**F**), and *R*_32_ (**G**).

Weighted multiple linear regression was employed to evaluate factor effects on the response variables (Equation (S5)). The fitted models for all six responses showed high *r*^2^ values, with residuals exhibiting a normal distribution and no evident systematic deviation, indicating good model adequacy and predictive performance (Figure 4B–G). Statistical significance of model parameters was further assessed by ANOVA (Tables S4.1–S4.3), and the coefficient estimates of the 18 factors are presented in Figure 4A.

For the ISVs injury model, LEN concentration (*x*_12_), Danshen dosage (*x*_13_), and LEN exposure duration (*x*_14_) significantly affected both response variables. In the thrombosis model, PHZ concentration (*x*_21_), Danshen dosage (*x*_22_), and dosing sequence (*x*_25_) were identified as significant factors. In the vascular stenosis model, FeCl_3_ concentration (*x*_32_), Danshen dosage (*x*_33_), and Danshen treatment duration (*x*_35_) showed significant effects. Across the three models, Danshen dosage and modeling agent concentration (LEN, PHZ, FeCl_3_) were consistently identified as significant factors, indicating their critical roles in bioactivity evaluation. Based on the significance and magnitude of regression coefficients, the above nine parameters were defined as CBPPs for the respective assays and were subsequently subjected to further optimization within the AQbD framework.

#### 3.1.4. Optimization of CBPPs Based on CCD and I-Optimal Design

**Figure 5:**
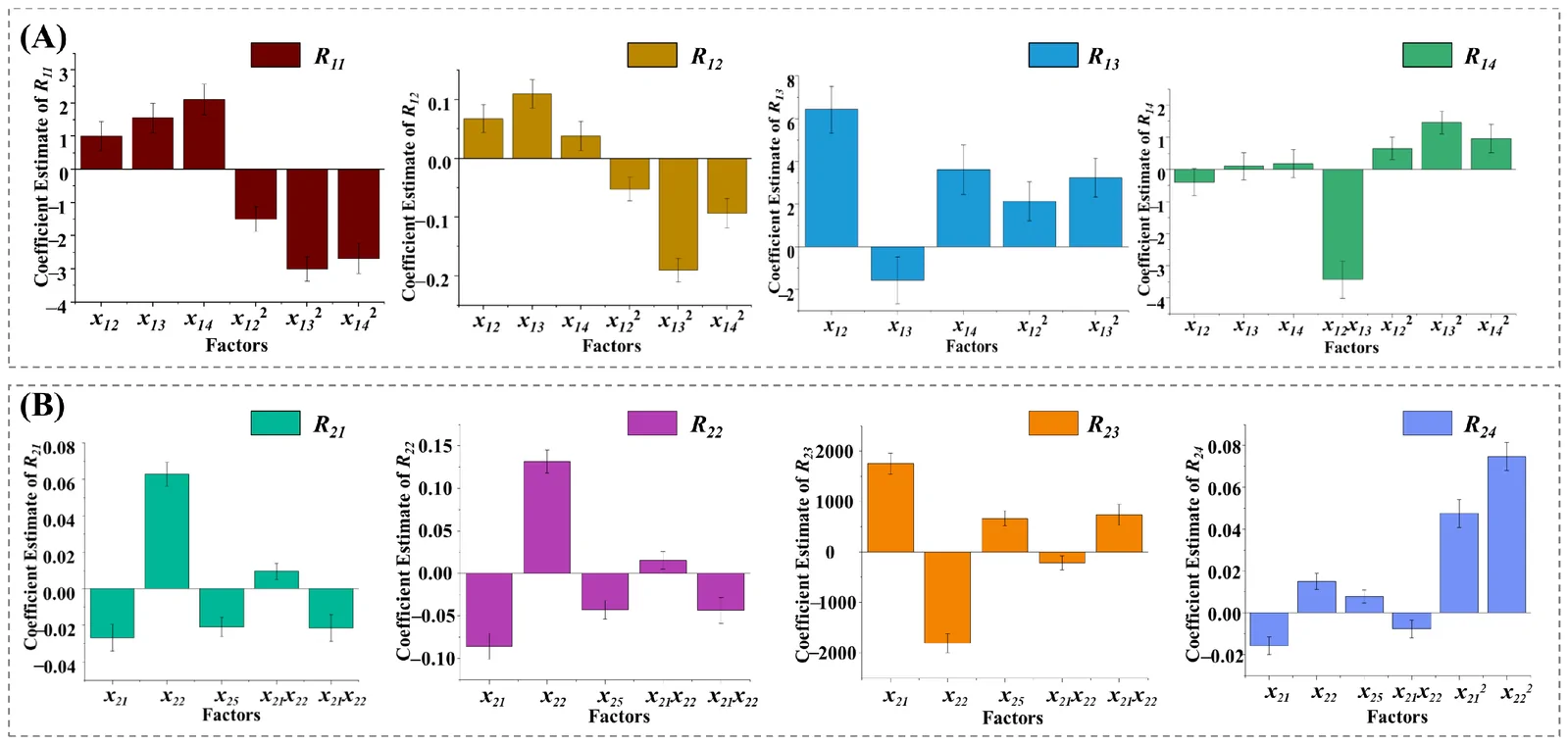

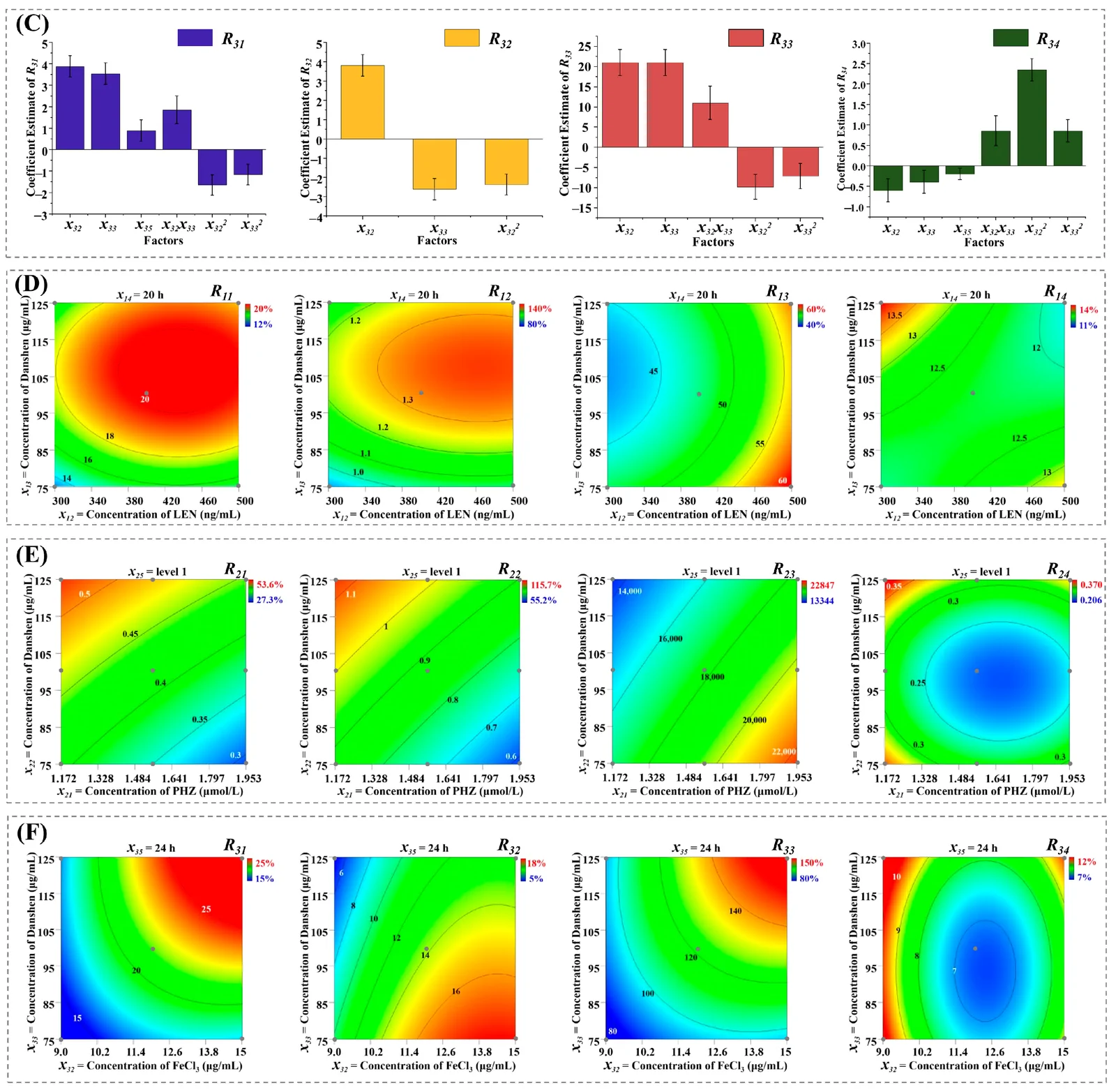
(**A**–**C**) Regression coefficients of the optimization models for pro-angiogenic (**A**), anti-thrombotic (**B**) and vasodilatory (**C**) assays; (**D**–**F**) Response surface plots of the optimization models for pro-angiogenic (**D**), anti-thrombotic (**E**) and vasodilatory (**F**) assays, with gray points representing experimental observations.

Following PB screening, the identified CBPPs for the three bioactivity assays were further optimized using response surface methodologies. The 12 CQAs listed in Table 1 were used as response variables for model optimization, among which the robustness indicators (*R*_14_, *R*_24_, *R*_34_) were expressed as coefficients of variation to reflect method reproducibility under varying conditions. For the pro-angiogenic and vasodilatory assays, CCD was applied, while an I-optimal design was employed for the anti-thrombotic assay due to the presence of a categorical factor (*x*_25_). A total of 57 experimental runs were conducted, including 20 runs for the pro-angiogenic assay, 17 runs for the anti-thrombotic assay, and 20 runs for the vasodilatory assay. The design matrices, factor levels, and experimental results are presented in Tables S5.1–S5.3. PLS regression models were established to describe the relationships between CBPPs and CQAs (Equation (S6)), with a significance threshold of *p* = 0.05 for term inclusion or exclusion. The results demonstrated significant nonlinear relationships between CBPPs and all response variables across the three assays. The regression coefficients and corresponding response surface plots are shown in Figure 5A–F. Model performance was evaluated using the coefficient of determination (r^2^), adjusted r^2^ (Adj r^2^), and predicted r^2^ (Pred r^2^), as summarized in Table S6. All models exhibited high goodness-of-fit, with particularly strong predictive performance observed in the anti-thrombotic assay (r^2^ > 0.93), followed by the vasodilatory (r^2^ > 0.87) and pro-angiogenic assays (r^2^ > 0.82). These results indicate that the established models are robust and suitable for subsequent design space construction and method optimization under the AQbD framework.

#### 3.1.5. Design Space Establishment

**Figure 6:**
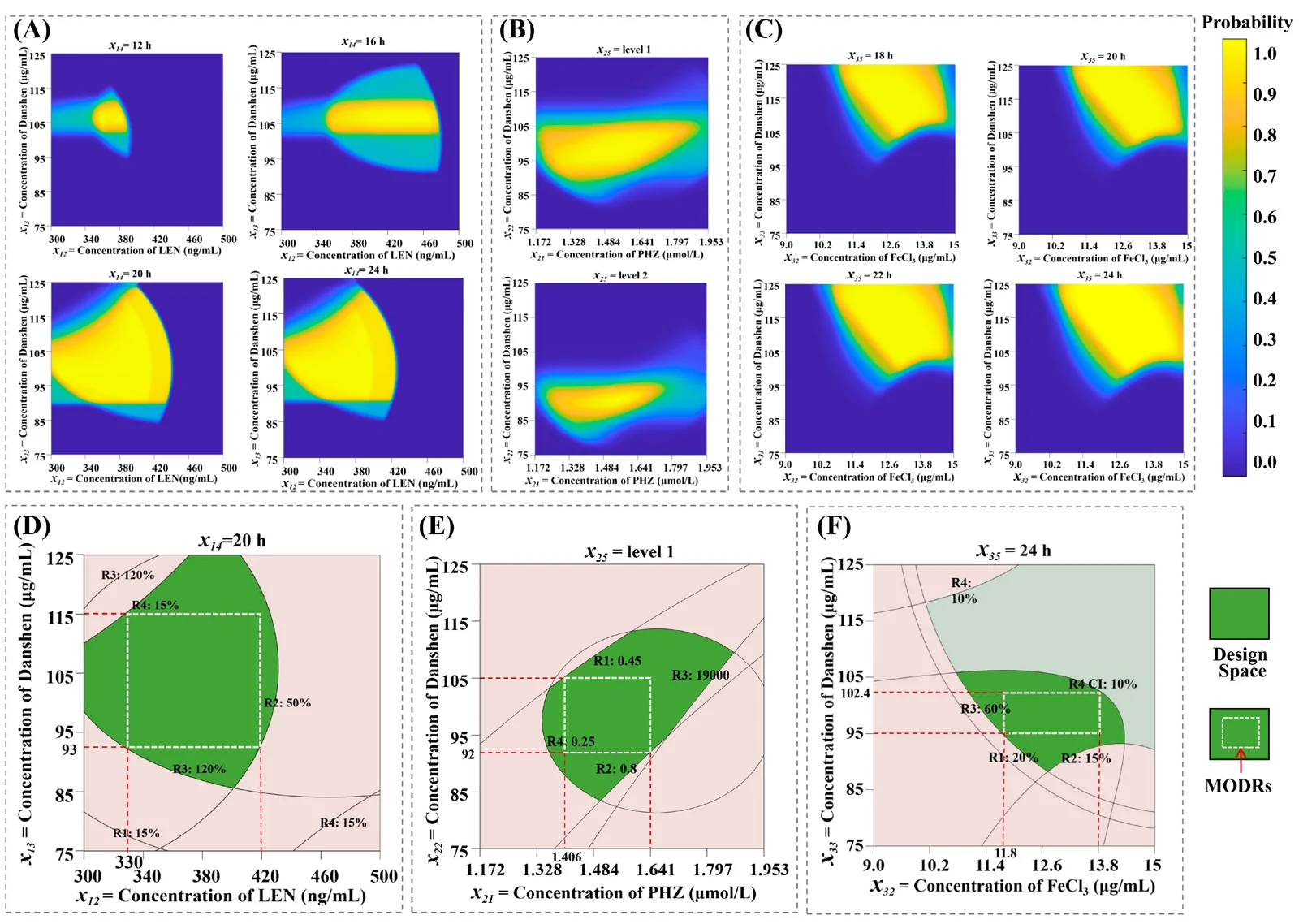
(**A**–**C**) Design spaces calculated by Monte Carlo simulation for the pro-angiogenic (**A**), anti-thrombotic (**B**), and vasodilatory (**C**) assays (the color gradient from blue to yellow represents an increasing probability (0–100%) of simultaneously meeting all CQA criteria under the corresponding parameter conditions); (**D**–**F**) MODRs (delineated by white dashed boxes) for the pro-angiogenic (**D**), anti-thrombotic (**E**), and vasodilatory (**F**) assays.

Design spaces for the three bioactivity assays were constructed based on the optimized AQbD models using deterministic constraints combined with Monte Carlo simulation. For each assay, two continuous CBPPs were treated as independent variables to construct two-dimensional design spaces, while a third CBPP was fixed at a representative level to assess its influence on system robustness. Monte Carlo simulation was applied to estimate the probability of simultaneously satisfying all CQAs, and the resulting design spaces are presented in Figure 6A–C. The fixed CBPP levels yielding the most robust design spaces were identified as *x*_14_ = 20 h for the pro-angiogenic assay, *x*_25_ = Level 1 for the anti-thrombotic assay, and *x*_35_ = 24 h for the vasodilatory assay, under which the design spaces were maximized and exhibited the highest probability of satisfying all CQAs. Based on the derived design spaces, two-dimensional projections were further visualized using linear programming to facilitate interpretation of the feasible regions (Figure 6D–F).

The MODRs were subsequently defined as practical subsets within the corresponding design spaces, ensuring experimental operability while maintaining a failure probability below 5%, which were determined as *x*_12_ = 330–420 ng/mL and *x*_13_ = 93–115 μg/mL (*x*_14_ = 20 h) for the pro-angiogenic assay; *x*_21_ = 1.406–1.641 μmol/L and *x*_22_ = 92–105 μg/mL (*x*_25_ = Level 1) for the anti-thrombotic assay; and *x*_32_ = 11.8–13.8 μg/mL and *x*_33_ = 95–102.4 μg/mL (*x*_35_ = 24 h) for the vasodilatory assay.

#### 3.1.6. Method Validation

Method validation was performed using 10 parameter combinations selected within the MODRs, including combinations located at the region boundaries. All results met the predefined CQA acceptance criteria, confirming assay robustness within the defined design space. Sensitivity to metabolite variability was further evaluated using Danshen samples from different origins. Significant differences in bioactivity were observed across all three assays (*p* < 0.05). Fisher’s multiple comparison results (Figure S2) further revealed distinct inter-sample variation patterns, indicating that the established methods can effectively capture bioactivity differences associated with metabolite composition variability. For subsequent modeling of metabolite–bioactivity relationships, a representative operating condition within the MODRs was selected for bioactivity data acquisition, defined as follows: *x*_12_ = 400 ng/mL, *x*_13_ = 100 μg/mL, and *x*_14_ = 20 h; *x*_21_ = 1.563 μmol/L, *x*_22_ = 100 μg/mL, *x*_25_ = Level 1, *x*_32_ = 12.5 μg/mL, *x*_33_ = 100 μg/mL, and *x*_35_ = 24 h.

### 3.2. Metabolite Characterization of Danshen Extracts Based on ^1^H NMR

**Figure 7:**
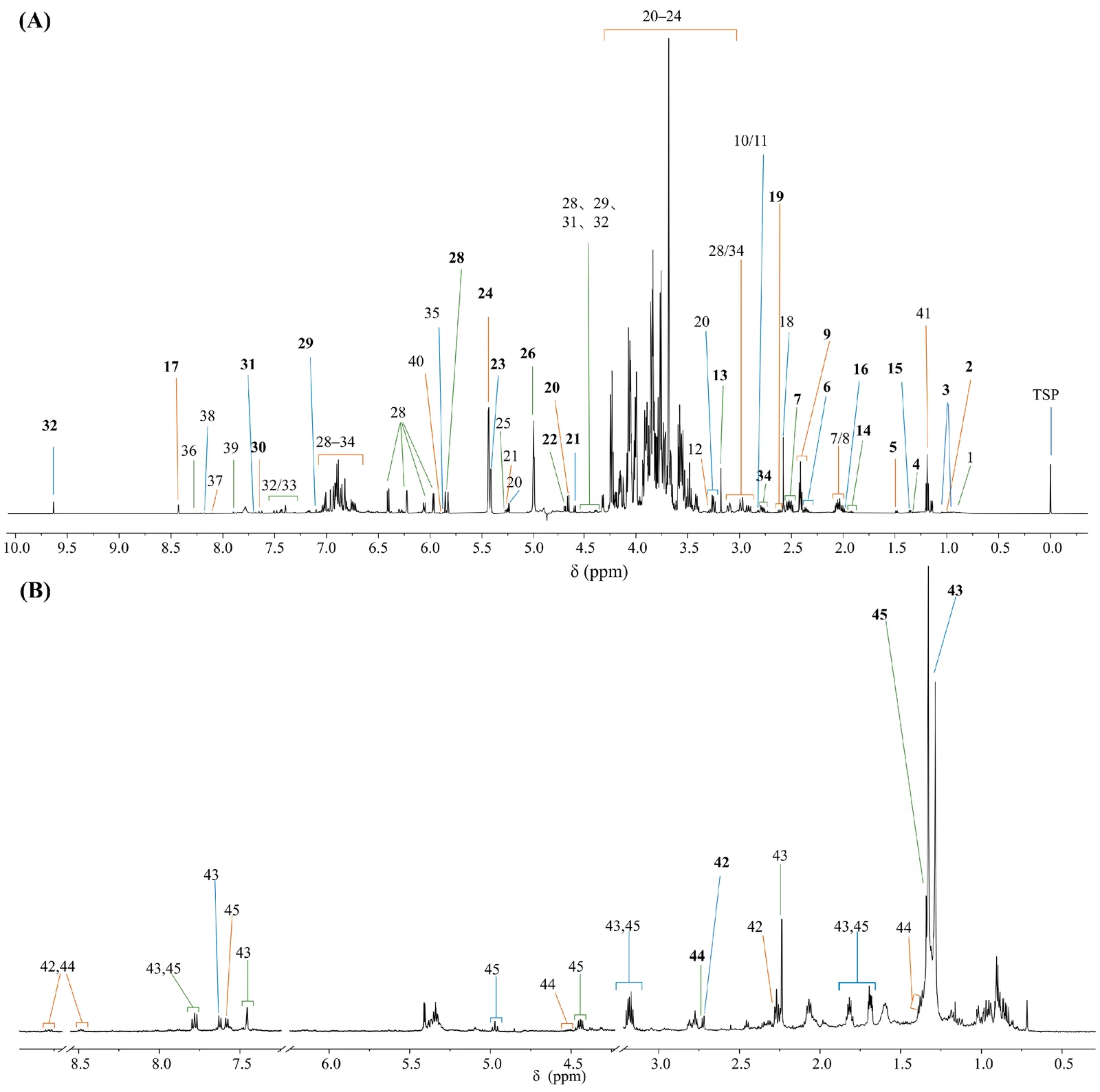
Representative ^1^H NMR spectra and peak assignments of water-soluble (**A**) and lipophilic (**B**) constituents of Danshen. Peaks: 1. Leucine (Leu); 2. Isoleucine (Iso); 3. Valine (Val); 4. Threonine (Thr); 5. Alanine (Ala); 6. Proline (Pro); 7. Glutamic acid (Glut); 8. Glutamine (Gln); 9. Pyroglutamic acid (Pyr); 10. Aspartic acid (Asp); 11. Asparagine (Asn); 12. Tryptophan (Try); 13. Malonic acid (Mal); 14. γ-Aminobutyric acid (GABA); 15. Lactic acid (Lac); 16. Acetic acid (Ace); 17. Formic acid (For); 18. Malic acid (Mali); 19. Succinic acid (Succ); 20. Glucose (Glu); 21. Galactose (Gal); 22. Fructose (Fru); 23. Sucrose (Sucr); 24. Raffinose (Raf); 25. Mannotriose (Man); 26. Stachyose (Sta); 27. 5-Hydroxymethylfurfural (5-HMF); 28. Salvianolic acid B (SaB); 29. Salvianolic acid A (SaA); 30. Rosmarinic acid (RA); 31. Lithospermic acid (LA); 32. Protocatechuic Aldehyde (PAH); 33. Protocatechuic acid (PA); 34. Salvianic acid A (DSS); 35. Uridine (Uri); 36. 2′-Deoxyadenosine (Deo); 37. Adenine (Ade); 38. Adenosine (Ado); 39. Guanosine (Gua); 40. Cytidine (Cyt); 41. Ethanol (Eth); 42. Tanshinone I (Tan I); 43. Tanshinone IIA (Tan IIA); 44. Dihydrotanshinone I (Dtan I); 45. Cryptotanshinone (Ctan).

To comprehensively characterize the metabolite composition of Danshen extracts, ^1^H NMR analyses were performed using 90% H_2_O /10% D_2_O and CD_3_OD as deuterated solvents to capture water-soluble and lipophilic constituents, respectively, yielding two complementary spectral profiles (Figure 7). The aqueous-phase spectra primarily reflected signals from primary metabolites (amino acids, nucleosides, organic acids, and sugars) as well as water-soluble secondary metabolites such as salvianolic acids, whereas the CD_3_OD spectra exhibited characteristic resonances of lipophilic metabolites, particularly tanshinones. The integration of these datasets enabled broad metabolite coverage.

Subsequently, spectral assignment was performed through systematic analysis, supported by literature data and reference standards, leading to the identification of 45 compounds (Table S7). Among these, 28 compounds exceeded the limit of quantification and showed well-resolved, non-overlapping signals, allowing reliable quantification (underlined and bold entries in Table S7). The number of reliably quantifiable metabolites was limited by signal overlap and low analyte concentrations, reflecting key challenges in the direct qNMR analysis of complex herbal extracts. A ^1^H qNMR method was applied to determine their concentrations across the 60 samples (Table S8), generating a multi-dimensional quantitative data matrix (X) for targeted metabolomics and machine learning analysis.

### 3.3. Construction of Multi-Activity Prediction Models for Danshen

To establish quantitative metabolite–bioactivity relationships, three normalized bioactivity indices were selected as response variables: relative rate of angiogenesis (Y_1_, *R*_13_), relative rate of thrombus resolution (Y_2_, *R*_22_), and relative rate of restoration (Y_3_, *R*_33_), which were determined across 60 Danshen samples (Table S9). As these indices were normalized against positive controls, they minimized inter-assay variability and more reliably reflected the corresponding cardiovascular bioactivities.

**Figure 8:**
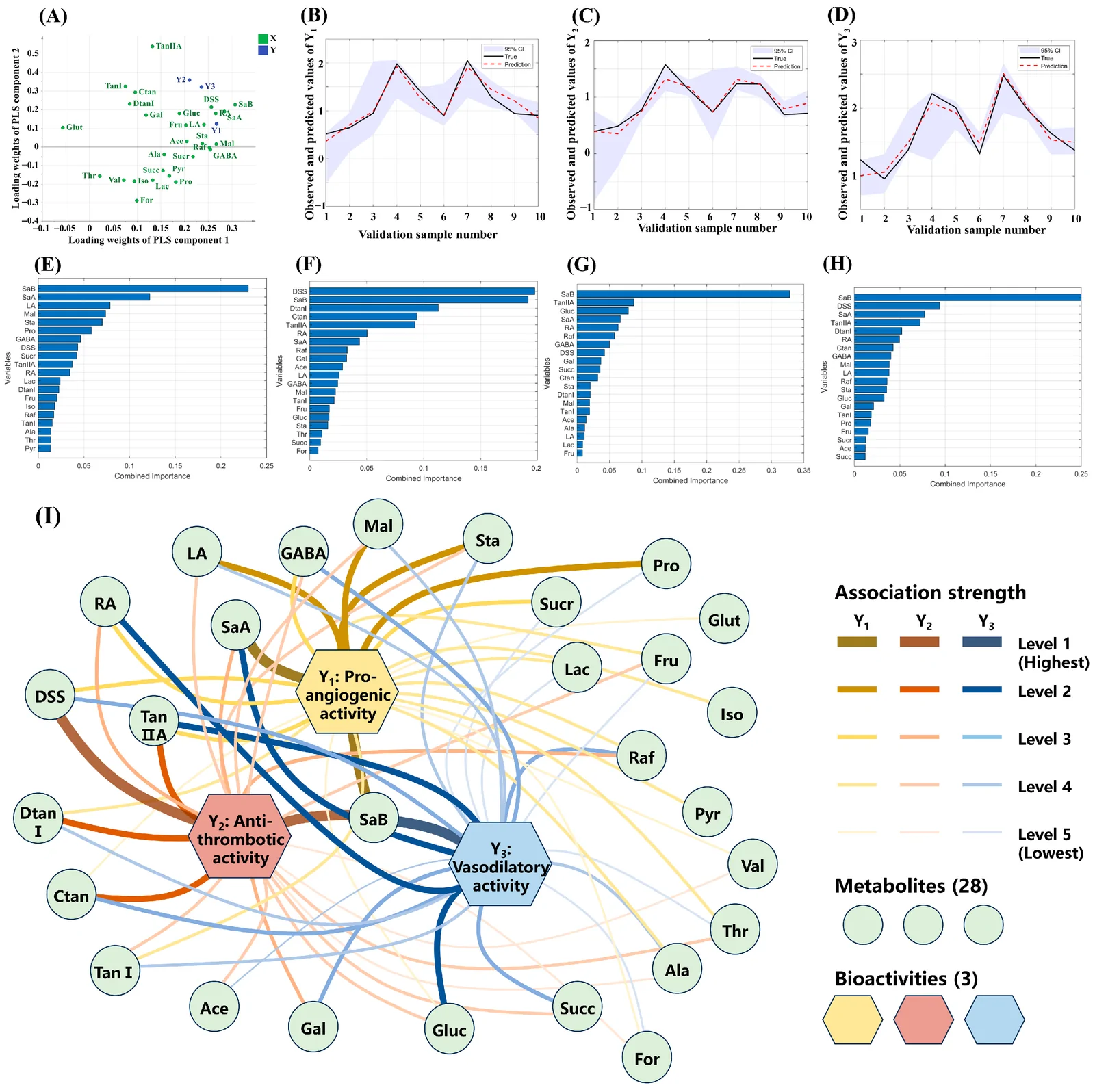
Metabolite–bioactivity relationship analysis and integrated network of Danshen. (**A**) Loading plots of PLS models for three bioactivities. (**B**–**D**) Uncertainty analysis of predicted results for pro-angiogenic (**B**), anti-thrombotic (**C**), and vasodilatory activity (**D**); (**E**–**G**) Variable importance rankings derived from stacked models for Y_1_ (**E**), Y_2_ (**F**), and Y_3_ (**G**); (**H**) Integrated variable importance across the three bioactivity models. (**I**) Metabolite–bioactivity relationships network.

PLS regression models were first constructed to evaluate linear relationships between the 28 quantified constituents and the three bioactivity responses. All models showed satisfactory performance, with R^2^ > 0.82 and Q^2^ > 0.61; detailed parameters are summarized in Table S10. Loading plots illustrated the overall associations between constituents and bioactivities, with shorter inter-variable distances indicating stronger correlations (Figure 8A). However, the limited predictive performance of the PLS models suggested potential nonlinear metabolite–bioactivity relationships.

To improve predictive performance, SVR, RF, and LSBoost were employed as base learners, and a stacking-based ensemble learning framework was developed to integrate their outputs and enhance prediction robustness. The contribution of each base model in the ensemble is summarized in Table S11. Model performance comparisons between individual learners and the stacked ensemble are presented in Table 2. The results indicate that the ensemble strategy consistently outperformed all single models, achieving higher predictive accuracy and improved stability across the three bioactivity endpoints. Accordingly, the stacked models were selected as the final prediction models for bioactivity estimation. External validation using 10 independent samples further confirmed the reliability of the models. Uncertainty analysis of the predicted results is shown in Figure 8B–D, demonstrating good agreement between predicted and observed values with acceptable confidence intervals.

## 4. Discussion

### 4.1. Overall Performance and Applicability of the Framework

The results presented above demonstrate that integrating quantitative ^1^H NMR metabolite profiling with lifecycle-oriented zebrafish bioassays and ensemble learning provides an effective approach for linking metabolite variation with multiple cardiovascular bioactivities in Danshen. The stacked models consistently showed better predictive performance than the individual learners across the three bioactivity endpoints, while cross-validation and independent external validation provided additional support for their predictive performance. Collectively, these findings indicate that the proposed framework can facilitate the integrated characterization of metabolite variation and multiple bioactivities, providing a practical approach for bioactivity-informed evaluation of complex herbal systems. Once established, the models may also facilitate the estimation of multiple bioactivities from metabolite profiles, thereby reducing the need for extensive bioactivity testing of additional samples. Nevertheless, the relatively small sample size in the present study may increase the risk of overfitting and limit the generalizability of the developed models. Further validation using larger and independently collected datasets from multiple sources will therefore be necessary to more comprehensively assess model robustness and generalizability.

### 4.2. Integration of the Multidimensional Metabolite–Bioactivity Relationships Network of Danshen

Variable importance rankings were used to assess the relative importance of individual metabolites for predicting the three bioactivities, and were subsequently integrated into a metabolite–bioactivity relationships network (Figure 8E–I). The network revealed both shared and activity-specific patterns of statistical importance underlying the cardiovascular bioactivities of Danshen. SaB consistently showed the highest or among the highest variable-importance scores across all three models, indicating that it represents a major common contributor to the pro-angiogenic, anti-thrombotic, and vasodilatory activities. SaA, RA, and DSS also exhibited relatively high contributions in multiple models, suggesting that phenolic acids constitute the core bioactive basis of Danshen cardiovascular activity.

Despite these shared characteristics, distinct contribution patterns were observed among the three bioactivities. The pro-angiogenic model showed relatively high contributions from several primary metabolites, including stachyose, Pro, GABA, and Mal. These findings suggest that angiogenic activity may be influenced not only by secondary metabolites, but also by metabolic pathways associated with energy metabolism, osmotic regulation, and cellular proliferation. In contrast, the anti-thrombotic model showed markedly increased contributions from lipophilic diterpenoid quinones, including Dtan I, Ctan, and Tan IIA, indicating that anti-thrombotic activity may rely on synergistic effects among water-soluble phenolic acids and lipophilic diterpenoids. For the vasodilatory model, SaB exhibited overwhelmingly dominant importance compared with other constituents, highlighting its central role in vasodilatory activity prediction. In addition, Tan IIA, RA, Raf, and Glu also contributed substantially, suggesting that vascular relaxation may involve coordinated regulation of endothelial protection, oxidative stress, and metabolic homeostasis.

Notably, multiple primary metabolites, including sugars, amino acids, and organic acids, exhibited non-negligible contributions across the three models. This finding indicates that the cardiovascular activities of Danshen cannot be fully explained by a few conventional marker compounds alone, but rather arise from coordinated effects of the overall metabolite network. Therefore, the established multi-dimensional metabolite–bioactivity relationships network provides a systems-level perspective for understanding the complex metabolite basis of Danshen bioactivity and further supports the necessity of multi-component and multi-bioactivity strategies for holistic quality evaluation of herbal medicines. Additionally, the above analysis reflects statistical associations and predictive relevance derived from the models, rather than direct evidence of biological causality or pharmacological action, and the identified metabolites should be considered candidate contributors requiring further experimental validation.

## 5. Conclusions

This study extended AQbD principles to zebrafish-based bioassay systems within a lifecycle-oriented strategy, enabling the systematic development of three cardiovascular bioassays for Danshen with well-defined design spaces and robust operating conditions. A combined ^1^H NMR fingerprinting and qNMR strategy enabled comprehensive and quantitative profiling of Danshen metabolites, generating a structurally resolved dataset suitable for integrative metabolomics–bioactivity modeling. An interpretable ensemble-learning framework was developed to establish quantitative metabolite–bioactivity relationships. The stacking ensemble improved predictive performance over individual machine-learning models and enabled efficient in silico prediction of multiple cardiovascular bioactivities from metabolite profiles. Network-based analysis further demonstrated that, beyond classical phenolic acids and tanshinones, multiple primary metabolites also showed substantial predictive importance across the cardiovascular bioactivity models. The resulting metabolite–bioactivity network revealed both shared and activity-specific metabolic contributors across the three bioactivities, highlighting a distinct pattern of shared and activity-specific predictive importance among metabolites.

Overall, this study establishes a lifecycle-oriented strategy for robust bioassay development and an interpretable ensemble-learning framework for decoding multi-component–multi-bioactivity relationships in complex herbal systems. By integrating quantitative metabolite profiling with robust multi-endpoint bioactivity measurements and nonlinear modeling, the framework enables bidirectional mapping between metabolite composition and multiple bioactivities, providing a potentially scalable and generalizable strategy for bioactivity-informed quality characterization and systems-level interpretation of herbal medicines.

## Figures and Tables

**Table 1:** Potential CBPPs and CQAs of the three bioactivity assays.

Pro-Angiogenic Assay (Model 1)		Anti-Thrombotic Assay (Model 2)		Vasodilatory Assay (Model 3)	
*x*_11_	Embryo developmental stage	*x*_21_	PHZ concentration	*x*_31_	Embryo developmental stage
*x*_12_	LEN concentration	*x*_22_	Danshen concentration	*x*_32_	FeCl_3_ concentration
*x*_13_	Danshen concentration	*x*_23_	PHZ exposure duration	*x*_33_	Danshen concentration
*x*_14_	LEN exposure duration	*x*_24_	Danshen treatment duration	*x*_34_	FeCl_3_ exposure duration
*x*_15_	Danshen treatment duration	*x*_25_	Dosing sequence	*x*_35_	Danshen treatment duration
*x*_16_	Dosing sequence	*x*_26_	Dechorionation status	*x*_36_	Dosing sequence
*R*_11_	Rate of angiogenesis	*R*_21_	Rate of thrombus resolution	*R*_31_	Restoration of vessel diameter
*R*_12_	Rate of ISV injury	*R*_22_	Relative rate of thrombus resolution	*R*_32_	Rate of vasoconstriction
*R*_13_	Relative rate of angiogenesis	*R*_23_	Thrombus area	*R*_33_	Relative rate of restoration
*R*_14_	Robustness of Model 1	*R*_24_	Robustness of Model 2	*R*_34_	Robustness of Model 3

**Table 2:** Cross-validation performance comparison of SVR, RF, LSBoost, and stacked models.

Models	Y_1_		Y_2_		Y_3_	
	R^2^	RMSE	R^2^	RMSE	R^2^	RMSE
SVR	0.91306	0.14538	0.79311	0.16298	0.50249	0.32671
RF	0.7215	0.26019	0.74285	0.18171	0.79412	0.21017
LSBoost	0.81625	0.21135	0.87975	0.12426	0.84167	0.18431
Stacking	0.9311	0.1295	0.8946	0.1163	0.9309	0.1218

## Data Availability

Further methodological details and supporting information are provided in the Supplementary Materials (Section S1: Literature-mining strategy, Section S2: Equations (S1)–(S7), Section S3: Tables S1–S11, and Section S4: Figures S1 and S2). The data underlying this article are available in the article and in its online Supplementary Materials.

## Supplementary Materials

The following supporting information can be downloaded at: https://www.mdpi.com/article/10.3390/metabo16100798/s1. Section S1: Literature-mining strategy; Section S2: Equations; Table S1: Batch numbers and origins of 60 batches of Danshen; Table S2: Computational parameters of the design space; Table S3.1: PB design matrices, factor coding values, and results for pro-angiogenic assay; Table S3.2: PB design matrices, factor coding values, and results for anti-thrombotic assay; Table S3.3: PB design matrices, factor coding values, and results for vasodilatory assay; Table S4.1: ANOVA of the PB screening model for the pro-angiogenic assay; Table S4.2: ANOVA of the PB screening model for the Anti-thrombotic assay; Table S4.3: ANOVA of the PB screening model for the vasodilatory assay; Table S5.1: CCD design matrices, factor coding values, and results for pro-angiogenic assay; Table S5.2: I-optimal design matrices, factor coding values, and results for anti-thrombotic assay; Table S5.3: CCD design matrices, factor coding values, and results for vasodilatory assay; Table S6: Goodness-of-fit metrics of three optimization models (r^2^, Adj r^2^, Pred r^2^, F-values, and *p*-values); Table S7: Assignment of Danshen 1H NMR fingerprint spectra; Table S8: ^1^H qNMR quantification of 28 metabolites in 60 batches of Danshen (mg/mL); Table S9: Normalized bioactivity indices (Y_1_–Y_3_) of 60 Danshen samples for spectrum–effect modeling; Table S10: Summary of Parameters for the Three PLS Models; Table S11: The contribution of base models (SVR, RF, LSBoost) in the ensemble of stacked model; Figure S1: (A) Preliminary range-finding of modeling agent concentrations; (B) Preliminary range-finding of Danshen concentrations; Figure S2: ANOVA–Fisher test results for the pro-angiogenic (A), anti-thrombotic (B), and vasodilatory (C) assays.

## Abbreviations

5-Hydroxymethylfurfural (5-HMF); Adenine (Ade); Adenosine (Ado); Acetic acid (Ace); Alanine (Ala); Analysis of variance (ANOVA); Analytical Quality-by-Design (AQbD); Asparagine (Asn); Aspartic acid (Asp); Critical bioassay procedure parameters (CBPPs); Central composite design (CCD); Methanol-d_4_ (CD_3_OD); Critical quality attributes (CQAs); Cryptotanshinone (Ctan); Cytidine (Cyt); Salvia miltiorrhiza Bunge (Danshen); 2′-Deoxyadenosine (Deo); Dimethyl sulfoxide (DMSO); Salvianic acid A (DSS); Dihydrotanshinone I (Dtan I); Deuterium oxide (D_2_O); Design of experiments (DoE); Ethanol (Eth); Formic acid (For); Fructose (Fru); γ-Aminobutyric acid (GABA); Galactose (Gal); Glucose (Glu); Glutamic acid (Glut); Glutamine (Gln); Guanosine (Gua); Hours post-fertilization (hpf); Intersegmental vessels (ISVs); Isoleucine (Iso); Lithospermic acid (LA); Lactic acid (Lac); Lenvatinib (LEN); Leucine (Leu); Gradient boosting (LSBoost); Malonic acid (Mal); Malic acid (Mali); Mannotriose (Man); Method operable design region (MODR); Out-of-fold (OOF); Protocatechuic acid (PA); Protocatechuic Aldehyde (PAH); Phenylhydrazine (PHZ); Partial least squares (PLS); 1-phenyl-2-thiourea (PTU); Pyroglutamic acid (Pyr); Quality markers (Q-markers); Rosmarinic acid (RA); Raffinose (Raf); Random forest (RF); Salvianolic acid A (SaA); Salvianolic acid B (SaB); Stachyose (Sta); Succinic acid (Succ); Sucrose (Sucr); Support vector regression (SVR); Tanshinone I (Tan I); Tanshinone IIA (Tan IIA); Threonine (Thr); Tetramethylsilane (TMS); 3-(trimethylsilyl) propionic-2,2,3,3-d_4_ acid sodium salt (TSP); Tryptophan (Try); Uridine (Uri); Valine (Val); quantitative ^1^H nuclear magnetic resonance (^1^H qNMR).
