# Supplementary material for: An Interpretable Ensemble-Learning Framework for Decoding Multi-Component–Multi-Bioactivity Relationships in Salvia miltiorrhiza Based on 1H NMR Metabolomics and Lifecycle-Oriented Bioassays
Source: Metabolites. 2026 Oct 21;16(10):798. doi: 10.3390/metabo16100798 (PMC13707060; doi:10.3390/metabo16100798)

Supplementary material

Title: An interpretable ensemble-learning framework for decoding multi-component–multi-bioactivity relationships in Salvia miltiorrhiza using ^{1}H NMR metabolomics and lifecycle-oriented bioassays

Authors: Wenzhu Li[a,b,c], Shasha Li[a], Hang Yu[a,b], Xiaozhou Zou[a], Yichao Lu[a,b], Zhiyan Chen[a,b], Nan Wang[a,b], Jiana Shi[a], Kaiyue Ji[*a], Ping Huang[*a]

Affiliation: a. Center for Clinical Pharmacy, Cancer Center, Department of Pharmacy， Zhejiang Provincial People's Hospital(Affiliated People's Hospital), Hangzhou Medical College, Hangzhou, Zhejiang, China

b. College of Pharmaceutical Sciences, Zhejiang University, Hangzhou 310058, China

c. Innovation Center in Zhejiang University, State Key Laboratory of Component-Based Chinese Medicine, Hangzhou 310058, China

***Correspondence:**

Ping Huang (huangping@hmc.edu.cn). Vice President, Zhejiang Provincial People's Hospital(Affiliated People's Hospital), Hangzhou Medical College, Hangzhou, 310014, China. ORCID: 0000-0003-1775-029X

Kaiyue Ji (jhyatt@163.com). Department of Pharmacy, Zhejiang Provincial People's Hospital (Affiliated People's Hospital), Hangzhou Medical College, Hangzhou 310014, Zhejiang, China

These authors contributed equally as corresponding authors

Contents

Section S1: Literature-mining strategy 3

Section S2: Equations 4

Equation S1: Calculations of angiogenesis-related indices 4
Equation S2: Calculations of thrombus-related indices 4
Equation S3: Calculations of vasodilation -related indices 4
Equation S4: ^{1}H NMR quantitative calculation 4
Equation S5: Weighted multiple linear regression 5
Equation S6: Partial least squares (PLS) regression models 5
Equation S7: Stacking ensemble model 5

Section S3: Tables 6

Table S1 Batch numbers and origins of 60 batches of Danshen 6
Table S2 Computational parameters of the design space 6
Table S3.1 PB design matrices, factor coding values, and results for pro-angiogenic assay 7
Table S3.2 PB design matrices, factor coding values, and results for anti-thrombotic assay 7
Table S3.3 PB design matrices, factor coding values, and results for vasodilatory assay 8
Table S4.1 ANOVA of the PB screening model for the pro-angiogenic assay 8
Table S4.2 ANOVA of the PB screening model for the Anti-thrombotic assay 9
Table S4.3 ANOVA of the PB screening model for the vasodilatory assay 9
Table S 5.1 CCD design matrices, factor coding values, and results for pro-angiogenic assay 10
Table S5.2 I-optimal design matrices, factor coding values, and results for anti-thrombotic assay ... 11
Table S5.3 CCD design matrices, factor coding values, and results for vasodilatory assay 12
Table S6 Goodness-of-fit metrics of three optimization models (r^2, Adj r^2, Pred r^2, F-values, and p-values) 13
Table S7. Assignment of Danshen ^{1}H NMR fingerprint spectra. 13
Table S8 ^{1}H qNMR quantification of 28 metabolites in 60 batches of Danshen (mg/mL). 17
Table S9. Normalized bioactivity indices (Y_1–Y_3) of 60 Danshen samples for spectrum–effect modeling 22
Table S10 Summary of Parameters for the Three PLS Models 23
Table S11 The contribution of base models (SVR, RF, LSBoost) in the ensemble of stacked model 23

Section S4: Figures 24

Figure S1. (A) Preliminary range-finding of modeling agent concentrations; (B) Preliminary range-finding of Danshen concentrations. 24
Figure S2. ANOVA–Fisher test results for the pro-angiogenic (A), anti-thrombotic (B), and vasodilatory (C) assays. 24

Section S1: Literature-mining strategy

Literature-mining strategy for cardiovascular-related bioactivities of Danshen

Pharmacological activity information for *Salvia miltiorrhiza* Bunge (Danshen) was collected through literature searches in PubMed and the China National Knowledge Infrastructure (CNKI). The literature published between January 1, 2016 and March 10, 2026 was searched using combinations of the terms "Danshen" or "Salvia miltiorrhiza" with terms related to cardiovascular pharmacological activities, zebrafish models, and clinical studies, including "zebrafish," "vasodilation," "vascular development," "anti-thrombotic," and "clinical," among others. After removal of duplicate records, the retrieved publications were reviewed to identify the major cardiovascular-related bioactivities reported for Danshen. The number of publications associated with each identified bioactivity was summarized and used to inform the selection of representative bioassay endpoints. Based on the literature findings and their relevance to the cardiovascular effects of Danshen, pro-angiogenic, anti-thrombotic, and vasodilatory activities were selected as representative endpoints for the present study.

Section S2: Equations

Equation S1: Calculations of angiogenesis-related indices

$$Rate\ of\ angiogenesis\ (R_{11}) = (\frac{L_{ID}}{L_{BD}} - \frac{L_{IM}}{L_{BM}})/\frac{L_{IC}}{L_{BC}} \times 100\%$$ Equation S1.1

$$Rate\ of\ ISVs\ Injury\ (R_{12}) = (\frac{L_{IC}}{L_{BC}} - \frac{L_{ID}}{L_{BD}})/\frac{L_{IC}}{L_{BC}} \times 100\%$$ Equation S1.2

$$Relative\ rate\ of\ angiogenesis\ (R_{13}) = R_{11}/(\frac{L_{IL}}{L_{BL}} - \frac{L_{IM}}{L_{BM}}) \times \frac{L_{IC}}{L_{BC}} \times 100\%$$ Equation S1.3

Where L_{ID}, L_{IL}, L_{IM}, and L_{IC}represent the ISVs length of the Danshen, LA, model, and vehicle control groups, respectively, and L_{BD}, L_{BM}, L_{BM}, and L_{BC} denote the corresponding body lengths of zebrafish in each group

Equation S2: Calculations of thrombus-related indices

$$Rate\ of\ thrombus\ resolution\ (R_{21}) = (A_M - A_D)/A_M \times 100\%$$ Equation S2.1

$$Relative\ rate\ of\ thrombus\ resolution\ (R_{22}) = R_{11}/(A_M - A_A)/A_M \times 100\%$$ Equation S2.2

Where A_D, A_A, and A_M represent the thrombus aera of the Danshen, aspirin, and model groups, respectively

Equation S3: Calculations of vasodilation -related indices

$$Restoration\ of\ vessel\ diameter\ (R_{31}) = (D_D - D_M)/D_C \times 100\%$$ Equation S3.1

$$Rate\ of\ vasoconstriction\ (R_{32}) = (D_C - D_D)/D_C \times 100\%$$ Equation S3.2

$$Relative\ rate\ of\ restoration\ (R_{33}) = R_{31}/(D_{DSS} - D_M) \times D_C \times 100\%$$ Equation S3.3

Where D_D, D_{DSS}, D_M, and D_C represent the caudal artery diameter of the Danshen, DSS, model, and vehicle control groups, respectively

Equation S4: ^{1}H NMR quantitative calculation

$$C_x = \frac{A_x}{A_I}\frac{N_I}{N_x}\frac{M_x}{M_I}C_I$$ Equation S4

Where C_x and C_I represent the mass concentrations of the analyte and the internal standard, respectively; A_x and A_I denote the characteristic peak areas of the analyte and the internal standard, respectively; N_x

and N_I correspond to the number of protons contributing to the characteristic signals of the analyte and the internal standard, respectively; and M_x and M_I represent the relative molecular weights of the analyte and the internal standard, respectively.

Equation S5: Weighted multiple linear regression

$$R = a_0 + \sum_{i=1}^{6} a_i x_i \qquad \text{Equation S5}$$

Where a_0 is the intercept term, and x_i and a_i represent the coded level of the corresponding analytical factor and its partial regression coefficient, respectively.

Equation S6: Partial least squares (PLS) regression models

$$R = b_0 + \sum_{i=1}^{3} b_i X_i + \sum_{i=1}^{3} b_{ii} {X_i}^2 + \sum_{i=1}^{2} \sum_{i=1}^{3} b_{ij} X_i X_j \qquad \text{Equation S6}$$

Where b_0 is the constant term, b_i, b_{ii}, and b_{ij} are regression coefficients, R is the response variable representing each evaluation index in the optimization experiment, and X_i and X_j are the coded values of the factors.

Equation S7: Stacking ensemble model

$$y = \beta_0 + \beta_1 y_{SVR} + \beta_2 y_{RF} + \beta_3 y_{LSBoost} \qquad \text{Equation S7}$$

where y represents the predicted value generated by the stacking ensemble model; y_{SVR}, y_{RF}, and $y_{LSBoost}$ denote the predicted outputs from the support vector regression (SVR), random forest (RF), and least-squares boosting (LSBoost) base models, respectively. β_0 is the intercept term, while β_1, β_2, and β_3 are the learned regression coefficients representing the relative contributions (weights) of each base learner in the final ensemble prediction.

Section S3: Tables

Table S1 Batch numbers and origins of 60 batches of Danshen

Batch No.	Origins	Batch No.	Origins	Batch No.	Origins
DS2401	Shandong province	DS2421	Sichuan province	DS2441	Henan province
DS2402	Shandong province	DS2422	Anhui province	DS2442	Henan province
DS2403	Shandong province	DS2423	Anhui province	DS2443	Henan province
DS2404	Shandong province	DS2424	Anhui province	DS2444	Henan province
DS2405	Shandong province	DS2425	Anhui province	DS2445	Henan province
DS2406	Shandong province	DS2426	Shanxi province	DS2446	Henan province
DS2407	Shandong province	DS2427	Shanxi province	DS2447	Sichuan province
DS2408	Shandong province	DS2428	Shaanxi province	DS2448	Sichuan province
DS2409	Yunnan province	DS2429	Shaanxi province	DS2449	Sichuan province
DS2410	Yunnan province	DS2430	Guizhou province	DS2450	Sichuan province
DS2411	Henan province	DS2431	Hebei province	DS2451	Anhui province
DS2412	Henan province	DS2432	Shandong province	DS2452	Anhui province
DS2413	Henan province	DS2433	Shandong province	DS2453	Anhui province
DS2414	Henan province	DS2434	Shandong province	DS2454	Anhui province
DS2415	Henan province	DS2435	Shandong province	DS2455	Shanxi province
DS2416	Henan province	DS2436	Shandong province	DS2456	Shanxi province
DS2417	Sichuan province	DS2437	Shandong province	DS2457	Shaanxi province
DS2418	Sichuan province	DS2438	Shandong province	DS2458	Shaanxi province
DS2419	Sichuan province	DS2439	Yunnan province	DS2459	Guizhou province
DS2420	Sichuan province	DS2440	Yunnan province	DS2460	Hebei province

Table S2 Computational parameters of the design space

Acceptable ranges for CMAs					
Responses	Limitations	Responses	Limitations	Responses	Limitations
R_{11}	≥15%	R_{21}	⩾45 %	R_{31}	≥20 %
R_{12}	⩽50%	R_{22}	⩾80 %	R_{32}	≤15%
R_{13}	≥120%	R_{23}	⩽19000mm^2	R_{33}	≥60%
R_{14}	≤15%	R_{24}	⩽25%	R_{34}	≤10%
**Monte Carlo simulation parameters**					
Number of simulations:	10000	Resolution:	64	Failure probability:	5%

Table S3.1 PB design matrices, factor coding values, and results for pro-angiogenic assay

No.	x_{11} (h)	x_{12} (ng/mL)	x_{13} (μg/mL)	x_{14} (h)	x_{15} (h)	x_{16}	R_{11}(%)	R_{12}(%)
1	48(+1)	500(+1)	75(-1)	24(+1)	24(+1)	2(+1)	12.68	57.34
2	24(-1)	500	125(+1)	12(-1)	24	2	16.07	53.96
3	48	300(-1)	125	24	12(-1)	2	7.67	47.19
4	24	500	75	24	24	1(-1)	11.18	58.84
5	24	300	125	12	24	2	11.06	43.81
6	24	300	75	24	12	2	4.10	50.76
7	48	300	75	12	24	1	8.24	46.63
8	48	500	75	12	12	2	13.25	56.78
9	48	500	125	12	12	1	15.88	54.15
10	24	500	125	24	12	1	17.01	53.02
11	48	300	125	24	24	1	8.24	46.63
12	24	300	75	12	12	1	7.86	47.00

Table S3.2 PB design matrices, factor coding values, and results for anti-thrombotic assay

No.	x_{21} (mmol/L)	x_{22} (μg/mL)	x_{23} (μg/mL)	x_{24} (h)	x_{25} (h)	x_{26}	R_{21} (%)	R_{22} (%)
13	1.953(+1)	125(+1)	18(−1)	24(+1)	2(+1)	2(+1)	16.91	35.25
14	1.172(−1)	125	24	18(−1)	2	2	34.05	65.65
15	1.953	75(−1)	24	24	1(−1)	2	37.01	71.37
16	1.172	125	18	24	2	1(−1)	32.55	67.86
17	1.172	75	24	18	1	2	51.86	100.00
18	1.172	75	18	24	1	2	44.07	91.85
19	1.953	75	18	18	2	1	31.76	66.20
20	1.953	125	18	18	1	2	18.77	39.11
21	1.953	125	24	18	1	1	38.64	74.51
22	1.172	125	24	24	1	1	37.01	71.37
23	1.953	75	24	24	2	1	30.99	59.76
24	1.172	75	18	18	1	1	49.48	103.14

Table S3.3 PB design matrices, factor coding values, and results for vasodilatory assay

No.	x_{31} (h)	x_{32} (μg/mL)	x_{33} (μg/mL)	x_{34} (h)	x_{35} (h)	x_{36}	R_{31} (%)	R_{32} (%)
25	72(+1)	15(+1)	125(+1)	18(−1)	24(+1)	2(+1)	12.9	20.2
26	72	9(−1)	125	24	18(−1)	2	20.5	14.8
27	48(−1)	15	75(−1)	24	24	2	17.4	2.9
28	72	9	125	18	24	1(−1)	14.5	19.1
29	72	9	75	24	18	2	16.3	3.8
30	48	9	75	18	24	2	11.4	7.9
31	72	15	75	18	18	1	8.1	10.6
32	48	15	125	18	18	2	5.0	25.8
33	48	15	125	24	18	1	20.8	14.6
34	48	9	125	24	24	1	22.5	13.4
35	72	15	75	24	24	1	17.1	3.1
36	48	9	75	18	18	1	8.1	10.6

Table S4.1 ANOVA of the PB screening model for the pro-angiogenic assay

Factors	R_{11}		R_{12}	
	F Value	P Value	F Value	P Value
x_{11}	0.074	0.7969	0.074	0.7969
x_{12} ***	64.43	0.0005	115.38	0.0001
x_{13}***	14.74	0.0121	14.74	0.0121
x_{14}**	5.60	0.0643	5.60	0.0643
x_{15}	0.12	0.7413	0.12	0.7413
x_{16}	0.54	0.4944	0.54	0.4944
r^2	0.9448		0.9647	
Adj r^2	0.8785		0.9222	
Pred r^2	0.6818		0.7964	

Table S4.2 ANOVA of the PB screening model for the Anti-thrombotic assay

Factors	R_{21}		R_{22}	
	F Value	P Value	F Value	P Value
x_{21}***	19.0285	0.0073	19.6144	0.0068
x_{22}***	15.0308	0.0117	15.6696	0.0108
x_{23}	3.6379	0.1148	0.7384	0.4294
x_{24}	1.6264	0.2582	1.5165	0.2729
x_{25}**	4.1462	0.0973	4.1221	0.0981
x_{26}	2.2131	0.1970	2.5609	0.1704
r^2	0.917		0.9137	
Adj r^2	0.8175		0.8101	
Pred r^2	0.4937		0.4761	

Table S4.3 ANOVA of the PB screening model for the vasodilatory assay

Factors	R_{31}		R_{32}	
	F Value	P Value	F Value	P Value
x_{31}	2.35	0.1856	2.31	0.1886
x_{32}***	5.23	0.071	189.75	< 0.0001
x_{33}***	49.3	0.0009	68.3	0.0004
x_{34}	0.29	0.6154	0.49	0.5155
x_{35}***	4.78	0.0806	7.4	0.0417
x_{36}	0.95	0.3736	0.62	0.4657
r^2	0.9264		0.9817	
Adj r^2	0.838		0.9598	
Pred r^2	0.5758		0.8949	

Table S 5.1 CCD design matrices, factor coding values, and results for pro-angiogenic assay

No.	Design matrices and factor coding values			Results			
	x_{12} (ng/mL)	x_{13} (μg/mL)	x_{14} (h)	R_{11} (%)	R_{12} (%)	R_{13} (%)	R_{14} (%)
1	300(−1)	75(−1)	12(−1)	8.318	46.549	79.811	13.660
2	500(+1)	75	12	13.141	56.884	95.957	14.497
3	300	125(+1)	12	10.385	44.481	99.646	19.279
4	500	125	12	15.799	54.227	115.364	11.493
5	300	75	24(+1)	11.320	50.092	88.207	11.325
6	500	75	24	14.618	57.475	110.924	20.597
7	300	125	24	14.864	46.549	115.820	20.149
8	500	125	24	17.866	54.227	135.574	10.568
9	220(−α)	100(0)	18(0)	14.950	38.280	101.504	13.732
10	600(+α)	100	18	13.289	72.831	113.565	14.851
11	400(0)	55(−α)	18	6.386	61.314	42.611	16.817
12	400	145(+α)	18	10.520	57.180	70.197	18.002
13	400	100	8(−α)	6.251	32.078	99.413	15.156
14	400	100	28(+α)	16.833	57.770	94.803	14.371
15	400	100	18	19.675	48.025	131.281	14.235
16	400	100	18	19.970	47.730	133.251	12.235
17	400	100	18	19.380	48.320	129.310	11.314
18	400	100	18	19.380	48.320	129.310	11.776
19	400	100	18	20.069	47.631	133.908	11.621
20	400	100	18	19.793	47.907	132.069	11.705

Table S5.2 I-optimal design matrices, factor coding values, and results for anti-thrombotic assay

No.	Design matrices and factor coding values			Results			
	x_{22} (μmol/L)	x_{23} (μg/mL)	x_{35}	R_{21} (%)	R_{22} (%)	R_{23} (mm^2)	R_{24} (%)
21	1.563(0)	75(−1)	1(−1)	38.402	80.024	18699.87	26.478
22	1.563	75	1	39.692	82.712	18308.28	26.748
23	1.953(+1)	75	1	40.215	81.232	18789.02	31.368
24	1.172(−1)	100(0)	1	44.387	95.836	15987.86	25.637
25	1.563	100	1	47.864	99.742	15827.38	20.579
26	1.953	100	1	46.243	93.407	17894.7	24.043
27	1.172	125(+1)	1	53.584	115.692	13343.98	36.748
28	1.563	125	1	51.270	106.839	14793.46	27.793
29	1.953	125	1	49.886	100.767	15749.61	34.356
30	1.172	75	2(+1)	41.965	90.607	16684.03	35.689
31	1.563	75	2	34.519	71.933	19878.63	28.946
32	1.953	75	2	27.304	55.152	22846.73	28.765
33	1.563	100	2	41.212	85.879	17846.87	22.228
34	1.563	100	2	44.664	93.073	16798.86	23.284
35	1.563	100	2	41.384	86.239	17794.46	22.496
36	1.172	125	2	50.279	108.558	14293.91	36.969
37	1.953	125	2	45.628	92.166	18087.8	34.458

Table S5.3 CCD design matrices, factor coding values, and results for vasodilatory assay

No.	Design matrices and factor coding values			Results			
	x_{32} (μg/mL)	x_{33} (μg/mL)	x_{35} (h)	R_{31} (%)	R_{32} (%)	R_{33} (%)	R_{34} (%)
38	9(−1)	75(−1)	12(−1)	10.109	8.942	58.739	13.562
39	15(+1)	75	12	13.528	19.765	78.091	11.620
40	9	125(+1)	12	16.355	3.777	95.029	10.341
41	15	125	12	26.760	10.413	154.470	8.505
42	9	75	24(+1)	11.074	8.144	64.345	9.645
43	15	75	24	15.563	18.327	89.834	10.719
44	9	125	24	16.938	3.295	98.415	12.104
45	15	125	24	29.305	8.614	169.160	8.792
46	7.2(−α)	100(0)	18(0)	10.111	3.059	64.194	13.715
47	16.8(+α)	100	18	23.379	14.367	133.302	12.419
48	12(0)	55(−α)	18	15.397	17.854	90.291	8.704
49	12	145(+α)	18	20.881	13.950	122.450	8.995
50	12	100	8(−α)	19.224	15.130	112.730	7.475
51	12	100	28(+α)	22.808	12.578	133.752	7.458
52	12	100	18	21.726	13.348	127.408	6.882
53	12	100	18	20.225	14.417	118.605	5.576
54	12	100	18	21.636	13.413	126.876	7.487
55	12	100	18	20.552	14.184	120.520	6.926
56	12	100	18	21.261	13.680	124.677	6.596
57	12	100	18	19.552	14.896	114.656	6.760

Table S6 Goodness-of-fit metrics of three optimization models (r^2, Adj r^2, Pred r^2, F-values, and p-values)

Responses	r^2	Adj r^2	Pred r^2	F Value	P Value
R_{11}	0.9045	0.8604	0.5118	20.52	< 0.0001
R_{12}	0.9025	0.8575	0.5149	20.06	< 0.0001
R_{13}	0.8209	0.7569	0.4535	12.83	< 0.0001
R_{14}	0.8256	0.7238	0.4636	8.11	0.0009
R_{21}	0.9353	0.8965	0.5936	24.11	< 0.0001
R_{22}	0.9432	0.9091	0.6429	27.66	< 0.0001
R_{23}	0.9550	0.9280	0.7580	35.35	< 0.0001
R_{24}	0.9670	0.9473	0.8717	48.89	< 0.0001
R_{31}	0.9138	0.8741	0.6339	22.98	< 0.0001
R_{32}	0.8464	0.8176	0.6838	29.39	< 0.0001
R_{33}	0.8852	0.8442	0.5923	21.6	< 0.0001
R_{34}	0.8770	0.8203	0.6074	15.46	< 0.0001

Table S7. Assignment of Danshen ^{1}H NMR fingerprint spectra.

No.	Chemical constituents	Characteristic chemical shifts and multiplicity of signals	Chemical structures
1	Leucine (Leu)[a]	0.96 (t, J = 6.0 Hz)	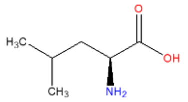
2	**Isoleucine (Iso)[a]**	**1.01 (d, J = 7.0 Hz)**, 0.94 (t)	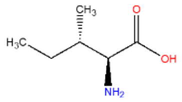
3	**Valine (Val)[a]**	**1.04 (d, J = 6.9 Hz)**, 0.99 (d, J = 6.9 Hz)	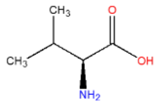
4	**Threonine (Thr)[a]**	**1.33 (d, J = 6.6 Hz)**	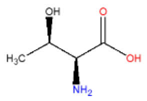

5	**Alanine (Ala)**[a]	**1.48 (d, *J* = 7.3 Hz)**	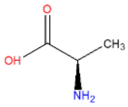
6	**Proline (Pro)**[a]	3.43 (m), 3.34 (m), **2.35 (m)**, 2.03 (m)	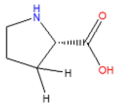
7	**Glutamic Acid (Glut)**[a]	3.80 (t, *J* = 6.3 Hz), **2.5(m)**, 2.1(m)	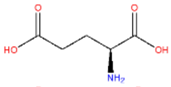
8	Glutamine (Gln)[a]	3.78 (t, *J* = 6.1 Hz), 2.1(m)	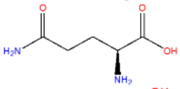
9	**Pyroglutamic Acid (Pyr)**[a]	2.05 (m), **2.42 (m)**, 2.51 (m)	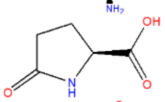
10	Aspartic Acid (Asp)[a]	4.05 (dd, *J* = 7.1, 4.5 Hz), 3.0 (m)	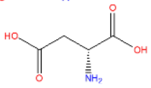
11	Asparagine (Asn)[a]	4.01 (dd, *J* = 9.4, 5.5 Hz)	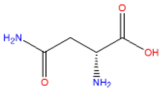
12	Tryptophan (Try)[a]	3.30 (dd, *J* = 15.6, 8.4 Hz)	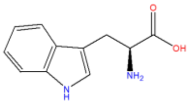
13	**Malonic Acid (Mal)**[a]	**3.17 (s)**	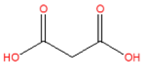
14	**γ- Aminobutyric Acid (GABA** [a]	**1.90 (quin)**	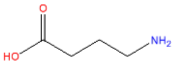
15	**Lactic Acid (Lac)**[a]	**1.40 (d, *J*=8.2 Hz)**	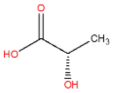
16	**Acetic Acid (Ace)**[a]	**1.97 (s)**	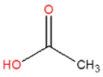
17	**Formic Acid (For)**[a]	**8.45 (s)**	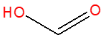
18	Malic Acid (Mali)[a]	2.63 (dd, *J* = 19.2, 5.0 Hz)	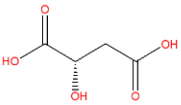
19	**Succinic Acid (Succ)**[a]	**2.59 (s)**	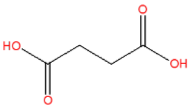
20	**Glucose (Glu)**[a]	**5.24 (d, *J* = 3.8 Hz), 3.25 (dd, *J* = 9.6, 8.0 Hz)**	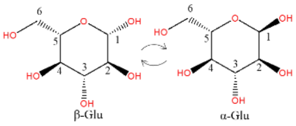
21	**Galactose (Gal)**[a]	**5.27 (d, *J* = 3.8 Hz), 4.59 (d, *J* = 7.9 Hz)**	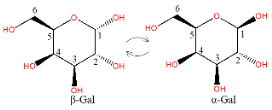

22	**Fructose (Fru)**[a]	**4.05 (m)**, 3.85 (m), 3.64 (m)	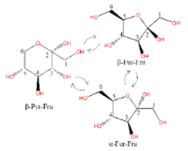
23	**Sucrose (Sucr)**[a]	**5.41 (d, *J* = 3.8 Hz)**, 4.22 (d, *J* = 8.8 Hz),	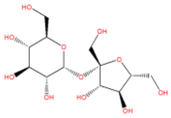
24	**Raffinose (Raf)**[a]	**5.43 (d, *J* = 3.9 Hz)**, 5.00 (d, *J* = 3.7 Hz), 4.23 (m, *J* = 8.8 Hz)	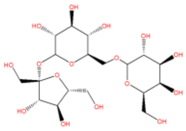
25	Mannotriose (Man)[a]	5.25 (d, *J* = 3.8 Hz), 4.65 (d, *J* = 8.0 Hz)	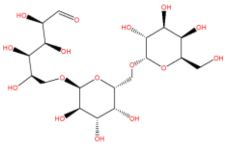
26	**Stachyose (Sta)**[a]	5.43 (d, *J* = 3.8 Hz), **4.99 (m)**, 4.23 (d, *J* = 8.8 Hz), 4.14 (dd, *J* = 7.7, 4.6 Hz)	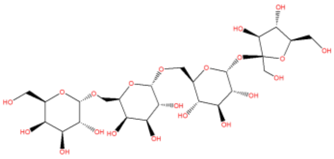
27	5-Hydroxymethylfurfural (5-HMF)[a]	9.44 (s)	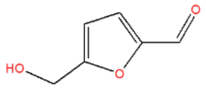
28	**Salvianolic Acid B (SaB)**[a]	6.41 (d, *J* = 8.1 Hz), 6.23 (d, *J* = 2.1 Hz), **6.06 (dd, *J* = 8.2, 2.1 Hz)**, 5.95 (d, *J* = 5.6 Hz), 5.84 (d, *J* = 16.2 Hz)	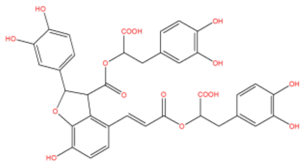
29	**Salvianolic Acid A (SaA)**[a]	7.05 (d, *J* = 1.9 Hz), 6.73 (d, *J* = 2.0 Hz), **6.56 (d, *J* = 8.5 Hz)**, 6.27 (d, *J* = 16.5 Hz)	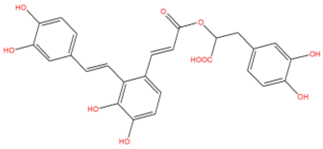
30	**Rosmarinic Acid (RA)**[a]	**7.45 (d, *J* = 16.2 Hz)**, 6.27 (d, *J* = 16.5 Hz)	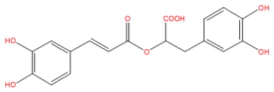
31	**Lithospermic Acid (LA)**[a]	**7.14 (d, *J* = 8.6 Hz)**, 6.28 (dd, *J* = 16.0, 0.9 Hz)	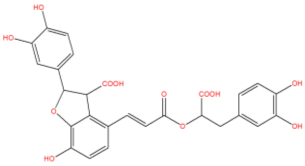
32	Protocatechuic Aldehyde (PAH)[a]	9.62 (s), 6.84 (m), 6.99 (d, *J* = 8.2 Hz)	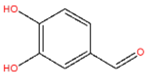
33	Protocatechuic Acid (PA)[a]	6.94 (d, *J* = 8.3 Hz)	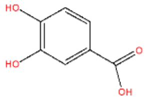
34	**Danshensu (DSS)**[a]	6.78 (d, *J*=2.1 Hz), **2.78 (dd, *J* = 14.1, 7.7 Hz)**	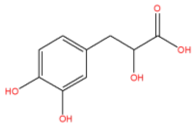

35	Uridine (Uri)[a]	7.86 (d, *J*=8.0 Hz), 5.90 (m)	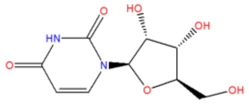
36	2′-Deoxyadenosine (Deo)[a]	8.20 (s), 8.30 (s)	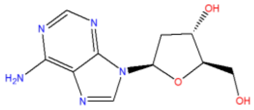
37	Adenine (Ade)[a]	8.21 (s), 8.24 (s)	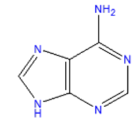
38	Adenosine (Ado)[a]	8.26 (s), 8.36 (s)	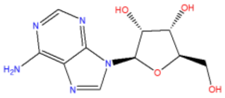
39	Guanosine (Gua)[a]	8.00 (s)	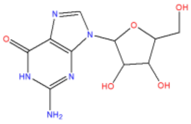
40	Cytidine (Cyt)[a]	5.87 (d, *J*=3.2 Hz)	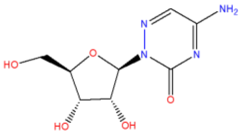
41	Ethanol (Eth)[a]	1.19 (t, *J* = 7.1 Hz)	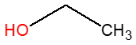
42	**Tanshinone I (Tan I)**[b]	9.29 (d, *J* = 8.8 Hz), 8.49 (dd, *J* = 8.8, 1.0 Hz), 7.42 (d, *J* = 7.1 Hz), **2.72 (s)**, 1.29 (s), 0.88 (s).	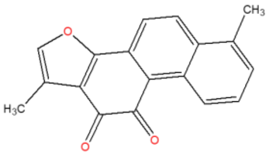
43	**Tanshinone IIA (Tan IIA)**[b]	7.78 (d, *J* = 8.1 Hz), 7.46 (q, *J* = 1.3 Hz), 3.17 (t, *J* = 6.4 Hz), 2.24 (d, *J* = 1.3 Hz), **1.33 (s).**	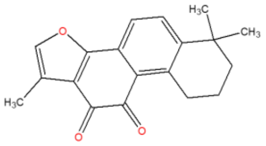
44	**Dihydrotanshinone I (Dtan I)**[b]	9.29 (d, *J* = 8.9 Hz), 8.48 (dd, *J* = 8.7, 1.0 Hz), 7.87 (d, *J* = 8.7 Hz), 5.05 (t, *J* = 9.6 Hz), **2.73 (s)**, 1.38 (d, *J* = 6.8 Hz)	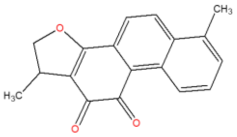
45	**Cryptotanshinone (Ctan)**[b]	7.79 (d, *J* = 8.1 Hz), 3.19 (t, J = 6.4 Hz), 1.82 (m), 1.69 (m), **1.34 (m)**	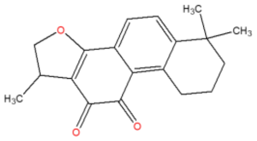

a Constituents assigned in the spectrum acquired using 90% H_2O + 10% D_2O as solvent (Fig. 7A).

b Constituents assigned in the spectrum acquired using MeOD as solvent (Fig. 7B).

Underlined and bolded entries indicate quantifiable constituents and their corresponding quantitative peaks.

Table S8 ^{1}H qNMR quantification of 28 metabolites in 60 batches of Danshen (mg/mL).

Batch No.	Iso	Val	Lac	Thr	Ala	GABA	Ace
1	0.0040	0.0085	0.0097	0.0058	0.0194	0.0371	0.0130
2	0.0026	0.0049	0.0024	0.0100	0.0150	0.0282	0.0218
3	0.0021	0.0065	0.0080	0.0104	0.0163	0.0267	0.0087
4	0.0014	0.0087	0.0059	0.0146	0.0225	0.0419	0.0180
5	0.0031	0.0083	0.0073	0.0199	0.0236	0.0515	0.0151
6	0.0025	0.0068	0.0037	0.0138	0.0201	0.0240	0.0091
7	0.0040	0.0041	0.0023	0.0256	0.0095	0.0113	0.0043
8	0.0135	0.0199	0.0133	0.0596	0.0357	0.0329	0.0214
9	0.0010	0.0059	0.0060	0.0137	0.0153	0.0183	0.0113
10	0.0015	0.0107	0.0172	0.0419	0.0320	0.0651	0.0386
11	0.0017	0.0082	0.0025	0.0230	0.0196	0.0212	0.0123
12	0.0021	0.0065	0.0057	0.0154	0.0210	0.0354	0.0114
13	0.0008	0.0056	0.0032	0.0116	0.0206	0.0303	0.0135
14	0.0055	0.0172	0.0031	0.0186	0.0172	0.0373	0.0271
15	0.0044	0.0093	0.0097	0.0125	0.0363	0.0374	0.0370
16	0.0280	0.0451	0.0107	0.0483	0.0367	0.0349	0.0088
17	0.0035	0.0090	0.0054	0.0138	0.0321	0.0276	0.0434
18	0.0039	0.0076	0.0076	0.0217	0.0607	0.0117	0.0114
19	0.0020	0.0013	0.0069	0.0076	0.0080	0.0109	0.0056
20	0.0018	0.0040	0.0062	0.0091	0.0147	0.0258	0.0287
21	0.0037	0.0094	0.0054	0.0491	0.0252	0.0329	0.0273
22	0.0055	0.0102	0.0083	0.0095	0.0165	0.0296	0.0090
23	0.0032	0.0096	0.0087	0.0081	0.0321	0.0351	0.0237
24	0.0056	0.0179	0.0106	0.0529	0.0298	0.0352	0.0298
25	0.0036	0.0107	0.0062	0.0042	0.0349	0.0193	0.0235
26	0.0057	0.0081	0.0096	0.0314	0.0201	0.0128	0.0082
27	0.0045	0.0080	0.0072	0.0161	0.0252	0.0215	0.0078
28	0.0040	0.0070	0.0041	0.0207	0.0172	0.0410	0.0050
29	0.0024	0.0057	0.0038	0.0179	0.0133	0.0288	0.0128
30	0.0020	0.0031	0.0062	0.0058	0.0247	0.0197	0.0313
31	0.0030	0.0116	0.0122	0.0324	0.0284	0.0178	0.0375
32	0.0114	0.0149	0.0068	0.0120	0.0266	0.0422	0.0171
33	0.0078	0.0097	0.0061	0.0172	0.0226	0.0416	0.0459
34	0.0229	0.0251	0.0189	0.0278	0.0410	0.0848	0.0303
35	0.0195	0.0125	0.0192	0.0254	0.0328	0.0624	0.0217
36	0.0090	0.0142	0.0116	0.0208	0.0344	0.0459	0.0096
37	0.0076	0.0290	0.0099	0.0495	0.0268	0.0430	0.0251
38	0.0177	0.0336	0.0200	0.1039	0.0557	0.0580	0.0270
39	0.0119	0.0152	0.0198	0.0370	0.0377	0.0785	0.0414
40	0.0085	0.0121	0.0159	0.0434	0.0490	0.0593	0.0381

41	0.0143	0.0130	0.0115	0.0422	0.0315	0.0335	0.0372
42	0.0145	0.0166	0.0129	0.0306	0.0391	0.0449	0.0236
43	0.0059	0.0101	0.0070	0.0110	0.0252	0.0334	0.0215
44	0.0093	0.0243	0.0090	0.0300	0.0256	0.0546	0.0384
45	0.0094	0.0174	0.0185	0.0177	0.0362	0.0595	0.0536
46	0.0636	0.1002	0.0224	0.0892	0.0760	0.0908	0.0379
47	0.0038	0.0111	0.0043	0.0223	0.0166	0.0190	0.0169
48	0.0053	0.0085	0.0142	0.1186	0.0227	0.0075	0.0092
49	0.0099	0.0160	0.0040	0.0319	0.0409	0.0698	0.0468
50	0.0081	0.0115	0.0096	0.0539	0.0339	0.0509	0.0279
51	0.0030	0.0068	0.0040	0.0100	0.0117	0.0164	0.0206
52	0.0034	0.0056	0.0053	0.0116	0.0165	0.0342	0.0149
53	0.0094	0.0250	0.0118	0.0816	0.0442	0.0698	0.0511
54	0.0138	0.0188	0.0367	0.0251	0.0537	0.1034	0.0743
55	0.0071	0.0236	0.0189	0.0587	0.0425	0.0458	0.0522
56	0.0050	0.0098	0.0033	0.0217	0.0339	0.0911	0.0325
57	0.0128	0.0211	0.0203	0.0420	0.0484	0.0878	0.0753
58	0.0071	0.0164	0.0168	0.0577	0.0413	0.0499	0.0530
59	0.0003	0.0024	0.0094	0.0093	0.0286	0.0246	0.0363
60	0.0124	0.0180	0.0088	0.0507	0.0424	0.0458	0.0189

Batch No.	Gln	Pro	Pyr	Succ	Mal	Raf	Fru
1	0.3924	0.1431	0.3215	0.0748	0.1207	1.4784	1.1126
2	0.2864	0.1028	0.4041	0.0609	0.1321	1.5825	1.7713
3	0.3069	0.1111	0.3818	0.0426	0.1248	1.1344	1.0914
4	0.3556	0.1573	0.4610	0.0738	0.1486	1.4651	1.8820
5	0.7334	0.1253	1.0379	0.0776	0.1344	1.3052	1.2259
6	0.3264	0.1040	0.4112	0.0652	0.1061	1.0323	1.0227
7	0.0738	0.0658	0.1882	0.0915	0.0965	1.3678	1.3995
8	0.1871	0.1064	0.7053	0.0766	0.0987	1.2460	1.4697
9	0.1788	0.0832	0.2782	0.0427	0.1047	0.6499	1.3355
10	0.3959	0.1928	0.7609	0.0784	0.2473	1.7459	2.6932
11	0.2628	0.1314	0.8296	0.0709	0.1283	1.1540	1.0458
12	0.3151	0.1539	0.7021	0.0603	0.1351	1.4904	1.6855
13	0.1397	0.1133	0.2819	0.0592	0.1414	1.3216	2.0676
14	0.3047	0.1148	0.2859	0.0528	0.1232	0.9495	1.0005
15	0.4439	0.1699	0.6187	0.0354	0.1493	1.4623	2.2460
16	0.5484	0.1345	0.1365	0.0621	0.0620	1.2049	0.2073
17	0.3349	0.1960	1.0097	0.0907	0.1539	1.6527	2.0366
18	0.0209	0.0299	0.2588	0.0924	0.0514	0.8975	2.5189
19	0.0333	0.0482	0.1797	0.0398	0.0394	0.7460	1.0080
20	0.1568	0.1525	0.3122	0.0898	0.1443	1.3091	2.1332
21	0.3830	0.2512	1.2801	0.0481	0.1381	1.3780	1.7547
22	0.4494	0.1309	0.9427	0.0835	0.1176	1.2201	1.0002

23	0.3086	0.1491	1.0427	0.0726	0.1317	1.9995	1.8954
24	0.3767	0.1530	0.6726	0.1275	0.1149	1.2630	1.3253
25	0.3025	0.0874	1.0452	0.0814	0.1327	1.6051	1.5080
26	0.1237	0.1248	0.7076	0.0554	0.1320	1.1207	1.7414
27	0.3200	0.1643	0.7960	0.0454	0.0996	1.1490	1.6780
28	0.5104	0.1010	0.9449	0.0369	0.1053	1.1740	1.2349
29	0.4022	0.1269	0.5930	0.0440	0.0939	0.8363	1.0048
30	0.2395	0.1241	0.5149	0.0202	0.1211	1.6218	1.9851
31	0.3120	0.2183	0.9368	0.1240	0.1278	1.3495	1.7213
32	0.0755	0.2305	0.0365	0.1322	0.1862	1.8915	1.6770
33	0.0684	0.1700	1.2472	0.0958	0.1807	2.0229	2.3493
34	0.1450	0.3204	2.1917	0.1846	0.3036	2.9361	2.7862
35	0.2002	0.2617	3.4961	0.0945	0.1922	2.2925	1.9100
36	0.0707	0.1878	1.5576	0.1138	0.1835	1.8328	1.8272
37	0.0655	0.1522	0.7129	0.1584	0.2275	2.3832	3.1386
38	0.0649	0.1743	1.5122	0.1263	0.1702	2.6831	2.4470
39	0.0629	0.2111	1.4597	0.0963	0.2708	2.3435	3.3118
40	0.0187	0.1183	1.3783	0.0783	0.2517	2.0881	3.0351
41	0.0509	0.2303	1.9402	0.0807	0.2215	2.1840	2.1347
42	0.1290	0.2699	1.9364	0.1350	0.2498	2.6455	3.1228
43	0.0067	0.1044	0.6418	0.1693	0.1781	1.5606	2.6827
44	0.1038	0.1916	1.1733	0.0970	0.1835	1.5216	1.6563
45	0.0738	0.2719	2.1211	0.1185	0.2707	2.5238	3.5021
46	0.2670	0.2884	2.4417	0.2399	0.1779	2.2577	0.5504
47	0.0282	0.1104	0.6804	0.0561	0.0929	0.8448	1.3069
48	0.0387	0.0954	0.4566	0.1493	0.0881	1.4336	3.6026
49	0.0437	0.2656	1.1361	0.1669	0.1733	2.9276	4.4981
50	0.1989	0.3196	2.7177	0.1469	0.2091	2.4491	2.6994
51	0.0661	0.0796	1.1447	0.0521	0.0891	0.7286	0.7329
52	0.0189	0.0799	1.0923	0.0472	0.1001	1.4848	1.2229
53	0.1616	0.1910	1.8441	0.2007	0.1886	2.3158	2.1885
54	0.2358	0.1318	3.2116	0.1088	0.3089	4.5744	3.7541
55	0.0819	0.2978	1.8479	0.1182	0.2288	2.2589	3.3267
56	0.0926	0.2760	1.7648	0.0805	0.1612	1.8961	2.4674
57	0.2311	0.2982	4.1645	0.0769	0.2773	3.5604	3.8214
58	0.1732	0.3382	3.4548	0.1387	0.2556	2.4787	1.9982
59	0.0155	0.1448	1.3327	0.0306	0.1857	2.2917	2.7330
60	0.1255	0.3287	1.9206	0.0913	0.1989	1.9933	2.8981

Batch No.	Sta	Glu	Gal	Sucr	SaB	DSS	SaA
1	22.9762	0.5363	0.2973	4.1061	3.4272	0.1670	0.2350
2	26.9670	0.9033	0.2907	2.6989	4.1117	0.0743	0.3068
3	21.9965	0.7189	0.1921	3.6043	3.9392	0.0883	0.3134
4	22.1578	1.1561	0.4846	4.0501	4.0313	0.0895	0.2814

5	24.4887	0.6965	0.2451	3.6678	4.6091	0.0659	0.3445
6	21.1160	0.6810	0.2492	2.6524	2.5159	0.0740	0.1937
7	10.9061	1.2051	0.5404	1.6730	1.3588	0.0430	0.0233
8	17.6176	1.1262	0.5881	2.5824	1.5442	0.0292	0.0468
9	9.5631	1.0395	0.5366	1.2687	2.6927	0.0293	0.1815
10	24.4084	2.2577	1.0046	3.1612	6.1000	0.1823	0.4308
11	20.3418	0.5179	0.2317	3.0343	3.7557	0.0141	0.2228
12	21.0823	0.9446	0.2765	2.8353	3.2822	0.0858	0.2473
13	14.7655	1.8912	0.6603	2.1868	4.3290	0.0508	0.3317
14	19.0499	0.8291	0.2531	2.8160	5.6497	0.0992	0.5207
15	25.6679	1.4823	0.6560	2.5744	4.1826	0.0421	0.1415
16	8.8029	0.1489	0.2205	2.0589	1.5511	0.0232	0.1706
17	21.2796	1.2314	0.3591	2.1616	4.2508	0.0549	0.2261
18	2.9284	1.5820	1.7577	2.0119	1.2468	0.1394	0.2626
19	5.4841	0.7706	0.6149	0.8233	1.5407	0.0161	0.0668
20	23.8180	1.3103	0.3114	2.0939	3.3688	0.0646	0.2298
21	22.9940	0.7588	0.3867	4.0141	1.9022	0.0348	0.1005
22	17.0929	0.6623	0.2155	2.5280	3.8096	0.1006	0.2290
23	19.1360	1.1774	0.8786	1.9623	4.0344	0.1669	0.2314
24	14.5303	0.9719	0.3178	1.9942	1.9982	0.0169	0.1770
25	19.1553	1.0737	0.8755	1.9800	4.3493	0.1375	0.1761
26	10.6387	0.9184	0.3486	1.7051	2.6450	0.0544	0.1858
27	17.4684	0.8506	0.3876	1.3695	2.7399	0.0838	0.1333
28	19.8138	0.9233	0.3431	1.8760	3.8127	0.0631	0.2694
29	14.8066	0.5089	0.1395	2.4397	2.1829	0.0358	0.1204
30	20.7524	1.3638	0.3643	1.0799	3.3181	0.0486	0.1687
31	13.0523	0.9050	0.3037	1.9645	2.8716	0.0624	0.2047
32	36.2383	0.9865	0.4232	6.7562	4.7938	0.1362	0.4046
33	38.7730	1.2181	0.3945	4.2597	5.3618	0.0545	0.4469
34	54.4781	1.6676	0.5046	8.2351	8.3167	0.1740	0.7011
35	36.8838	1.0122	0.3254	5.6161	6.1779	0.1678	0.4945
36	33.6919	1.0450	0.4056	4.4307	3.7577	0.1250	0.3065
37	24.0345	2.6744	1.1416	4.2617	2.6075	0.1544	0.1784
38	28.6460	1.6837	0.9464	4.3496	2.1542	0.0551	0.0985
39	22.2896	2.4528	1.1200	3.0753	6.0904	0.2256	0.4968
40	22.6788	2.0525	0.9052	2.9136	5.2102	0.2640	0.5371
41	33.3295	0.7944	0.2771	5.3931	5.1439	0.0765	0.4135
42	35.1754	1.4871	0.4404	4.6536	4.9849	0.1136	0.4602
43	15.6800	2.1041	0.9040	2.8332	4.3154	0.0759	0.4228
44	26.7768	1.1684	0.3199	4.2233	7.7334	0.1641	0.6589
45	36.8729	2.1047	1.0126	3.8823	5.4636	0.0705	0.3536
46	24.8511	0.4487	0.6089	7.2960	3.6914	0.0503	0.3561
47	9.1439	0.6541	0.2571	1.0916	1.8551	0.0233	0.1314
48	5.0882	2.6345	2.7889	0.3960	1.9035	0.0320	0.0407

49	28.2684	3.0525	2.5690	4.2287	6.7118	0.1654	0.3020
50	37.7201	1.4819	0.5251	7.8027	2.8827	0.0695	0.1229
51	13.1878	0.5037	0.1121	2.0478	2.4500	0.0193	0.1496
52	14.0293	0.7298	0.6782	1.3814	2.9140	0.0215	0.1578
53	28.3208	1.5958	0.5005	3.9466	3.0230	0.0285	0.2972
54	44.8035	2.4184	1.5546	4.9401	8.4651	0.0430	0.6831
55	19.6508	1.7302	0.5997	3.0508	4.6056	0.0996	0.3616
56	25.7333	1.3679	0.5076	3.0134	3.3029	0.0359	0.1947
57	56.2691	2.8154	0.8570	6.6138	9.2279	0.1254	0.7759
58	43.4412	1.5889	0.1749	7.2201	5.4649	0.0313	0.4036
59	26.1602	1.8925	0.4842	1.5855	4.1461	0.1239	0.2946
60	22.1539	1.4806	0.4651	3.1207	3.8429	0.0599	0.2218

Batch No.	RA	LA	For	Tan I	Dtan I	Tan IIA	Ctan
1	0.1505	0.0753	0.0053	0.2830	0.4244	3.9215	1.9105
2	0.1973	0.0960	0.0043	0.3241	0.4170	4.6663	2.2878
3	0.2013	0.0951	0.0042	0.3466	0.4764	3.6918	1.7532
4	0.1837	0.0856	0.0066	0.1875	0.3299	3.3091	2.1020
5	0.2542	0.1010	0.0048	0.1522	0.2345	6.1717	1.5867
6	0.1443	0.0600	0.0041	0.3297	0.5797	4.4107	1.5748
7	0.0596	0.0541	0.0057	0.5749	0.8386	7.9191	2.6333
8	0.0526	0.0533	0.0136	0.1657	0.2124	5.2181	2.2105
9	0.1400	0.0818	0.0022	0.1058	0.1573	2.9640	2.0691
10	0.2882	0.1728	0.0074	0.2220	0.2588	5.4064	3.7797
11	0.1715	0.1395	0.0123	0.1545	0.2153	3.1137	2.2821
12	0.1621	0.1098	0.0107	0.1576	0.1909	2.7989	2.1214
13	0.2185	0.1357	0.0058	0.2780	0.2109	4.1571	2.3277
14	0.3868	0.2845	0.0000	0.3851	0.4623	4.2463	1.6210
15	0.1458	0.0934	0.0034	0.1765	0.2782	3.9114	3.1336
16	0.1055	0.0433	0.0078	0.2747	0.4945	3.7205	1.7882
17	0.1768	0.1243	0.0037	0.0462	0.1380	2.6689	2.6765
18	0.0397	0.0123	0.0020	0.3121	0.1674	6.0088	4.9625
19	0.0572	0.0681	0.0042	0.1005	0.1335	2.9626	1.9293
20	0.1451	0.0810	0.0071	0.1610	0.1928	2.8913	2.4148
21	0.0598	0.1139	0.0159	0.1537	0.1918	5.1835	2.5412
22	0.1493	0.1459	0.0106	0.0761	0.1225	1.7576	0.5822
23	0.1551	0.1834	0.0031	0.1620	0.1947	5.1564	3.0850
24	0.1321	0.0585	0.0051	0.2572	0.1892	3.7868	2.3550
25	0.1627	0.2008	0.0021	0.1434	0.2134	3.7353	2.3811
26	0.1393	0.1670	0.0055	0.4656	0.3808	4.3077	2.5221
27	0.0978	0.1026	0.0112	0.1497	0.2300	3.4030	2.4288
28	0.1785	0.0938	0.0025	0.1843	0.2283	4.9038	1.5578
29	0.0884	0.0489	0.0059	0.1864	0.2254	5.1605	2.0121
30	0.1244	0.0735	0.0029	0.0409	0.0847	3.6996	2.8700

31	0.1204	0.1201	0.0171	0.4220	0.3484	3.3548	3.3790
32	0.2180	0.1015	0.0149	0.2289	0.2975	4.0334	1.9711
33	0.2856	0.1284	0.0145	0.2405	0.3353	4.0129	2.0400
34	0.5622	0.1984	0.0230	0.2702	0.3695	3.5478	2.0494
35	0.3279	0.1844	0.0148	0.1225	0.2146	4.8203	1.8315
36	0.2008	0.0974	0.0156	0.2228	0.4706	3.2897	2.0337
37	0.1480	0.1219	0.0232	0.4694	0.6687	6.5642	3.7943
38	0.0715	0.0901	0.0251	0.1196	0.1673	4.7859	2.0826
39	0.3140	0.1834	0.0077	0.1371	0.1906	4.0548	2.9116
40	0.2648	0.1507	0.0076	0.1131	0.1905	2.8818	2.1511
41	0.2903	0.2234	0.0313	0.0998	0.1521	2.7061	1.7387
42	0.2659	0.1786	0.0306	0.1467	0.1822	3.1174	2.3096
43	0.2676	0.1372	0.0097	0.1396	0.1222	2.2460	1.9328
44	0.6674	0.3006	0.0130	0.3370	0.4925	3.5940	2.1914
45	0.2247	0.1476	0.0091	0.0801	0.1479	2.5224	2.0107
46	0.2471	0.1360	0.0263	0.2354	0.6572	4.6996	2.5044
47	0.1050	0.1053	0.0100	0.1479	0.1111	2.2560	2.1040
48	0.0656	0.0851	0.0109	0.2414	0.3190	4.1716	4.4911
49	0.2077	0.2599	0.0310	0.1630	0.2079	3.4998	2.7298
50	0.0419	0.1408	0.0273	0.1302	0.1662	3.5981	2.2105
51	0.1021	0.1122	0.0106	0.0753	0.1381	2.2012	1.3904
52	0.1197	0.1285	0.0058	0.0703	0.0777	2.0338	1.4836
53	0.2148	0.1270	0.0240	0.1114	0.2085	2.4724	2.1118
54	0.4342	0.3434	0.0125	0.1908	0.2196	5.4738	3.1806
55	0.2692	0.2286	0.0291	0.3366	0.2999	3.2416	2.5123
56	0.1333	0.1141	0.0221	0.1024	0.1521	2.2106	1.9623
57	0.5421	0.2887	0.0180	0.2342	0.3010	7.3001	2.3796
58	0.2551	0.1759	0.0247	0.1323	0.2241	4.2126	1.3349
59	0.1900	0.1138	0.0064	0.0242	0.0570	1.3672	1.7496
60	0.1809	0.1293	0.0371	0.2314	0.2136	2.2600	2.2424

Table S9. Normalized bioactivity indices (Y_1–Y_3) of 60 Danshen samples for spectrum–effect modeling

No.	1	2	3	4	5	6	7	8	9	10
Y_1	0.9960	1.3895	1.1710	1.1745	1.5845	0.8034	1.5212	0.8721	0.6131	1.7148
Y_2	0.9096	1.2116	0.9323	0.9895	1.2622	0.7455	1.2443	0.8054	0.5893	1.3520
Y_3	1.6147	1.7591	1.5707	1.6425	1.9835	1.6026	0.8268	0.9963	1.1005	2.1515
No.	11	12	13	14	15	16	17	18	19	20
Y_1	0.7774	0.7060	1.5122	1.5581	1.3888	0.4703	1.0434	1.2830	0.4408	0.7864
Y_2	0.8687	0.7121	1.1770	1.2537	1.1895	0.5215	0.9978	1.2542	0.2728	0.8330
Y_3	1.4330	1.3414	1.5347	1.9982	1.7024	1.1511	1.4272	1.7808	0.9101	1.3372

No.	21	22	23	24	25	26	27	28	29	30
Y_1	0.8915	0.6002	1.5232	0.6666	1.3871	0.8963	0.6738	1.3893	0.8539	1.1387
Y_2	0.9244	0.4616	1.2566	0.6699	1.1252	0.9267	0.7355	1.1059	0.8733	1.0302
Y_3	1.3338	1.0639	1.8760	1.3590	1.4588	2.0634	1.1932	1.4356	1.3715	1.3329
No.	31	32	33	34	35	36	37	38	39	40
Y_1	0.7638	1.4359	1.5525	1.6883	1.5924	1.0379	1.5862	1.1853	1.6449	1.5099
Y_2	0.9584	1.1951	1.2533	1.3199	1.2722	0.8757	1.2704	0.8466	1.3003	1.1713
Y_3	1.4283	2.0855	1.7538	2.5970	2.3061	1.7061	1.6863	1.3647	2.0103	1.7081
No.	41	42	43	44	45	46	47	48	49	50
Y_1	1.2674	1.3941	1.2587	1.6095	1.4962	1.3431	0.3681	1.1990	1.6557	1.0032
Y_2	1.0791	1.1277	0.8533	1.3166	1.0791	1.1777	0.2340	1.1008	1.2720	0.7717
Y_3	1.9220	1.9502	1.3984	2.3709	1.9119	2.0866	1.1949	1.3431	2.3644	1.9852
No.	51	52	53	54	55	56	57	58	59	60
Y_1	0.5196	0.6489	0.9505	1.9789	1.4037	0.8958	2.0475	1.2886	0.9412	0.9075
Y_2	0.3893	0.4865	0.7750	1.5745	1.1270	0.7353	1.2355	1.2338	0.6906	0.7121
Y_3	1.2354	0.9572	1.3800	2.2096	2.0092	1.3264	2.4805	1.9797	1.6271	1.3752

Table S10 Summary of Parameters for the Three PLS Models

Response Variable	PC1 Explained Variance (%)	PC2 Explained Variance (%)	PC3 Explained Variance (%)	R^2	Q^2
Y_1	36.6	11.3	8.4	0.868	0.682
Y_2	35.9	11.4	8.1	0.851	0.612
Y_3	37.6	11.4	9.3	0.824	0.641

Table S11 The contribution of base models (SVR, RF, LSBoost) in the ensemble of stacked model

Y	SVR	RF	LSBoost
1	0.8873	0.0493	0.0634
2	0.1580	0.1332	0.7088
3	0.0133	0.3818	0.6049

Section S4: Figures

Figure S1. (A) Preliminary range-finding of modeling agent concentrations; (B) Preliminary range-finding of Danshen concentrations.

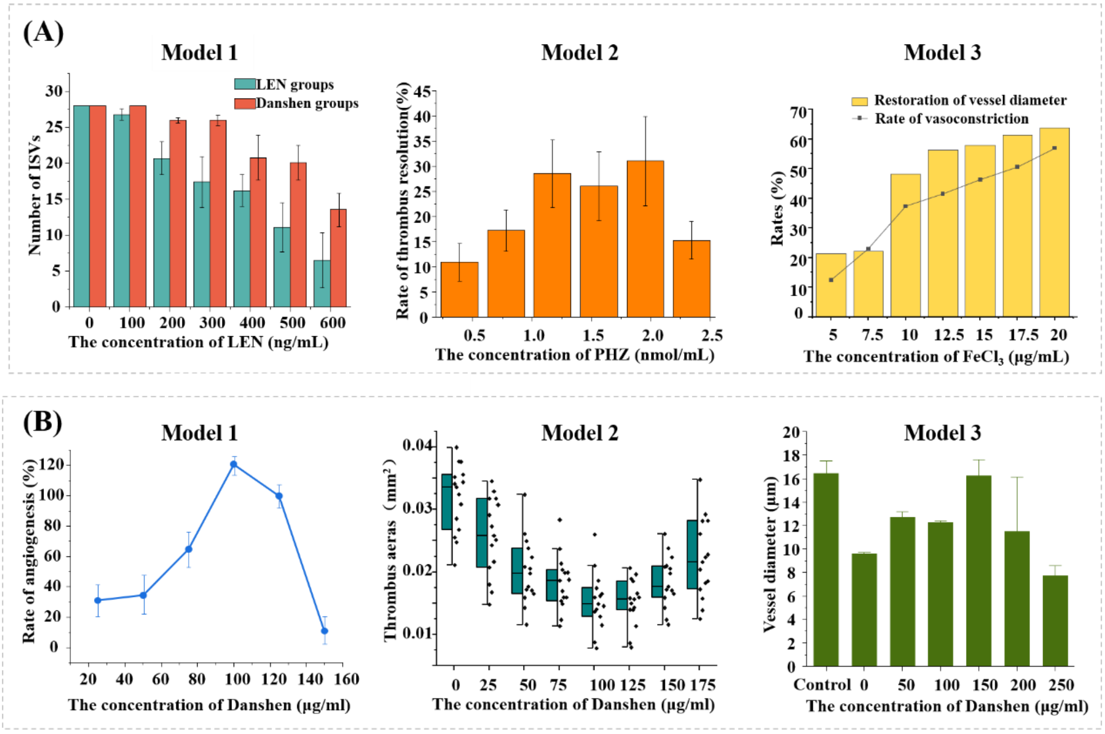

Figure S2. ANOVA–Fisher test results for the pro-angiogenic (A), anti-thrombotic (B), and vasodilatory (C) assays.

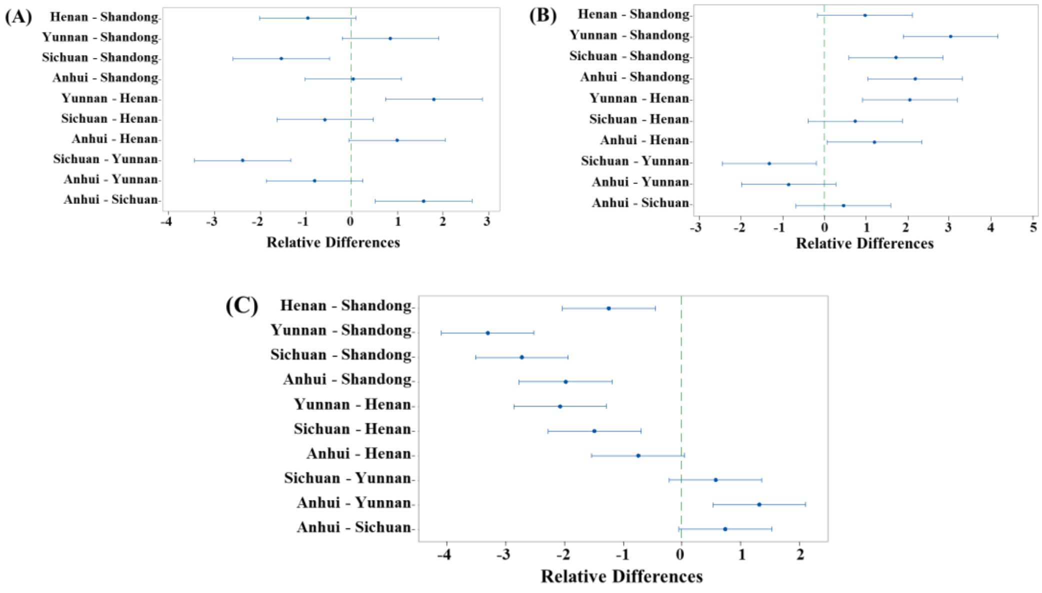

Supplement: Supplementary file 1 [file metabolites-16-00798-s001.zip › metabolites-4583205-supplementary.pdf]
